# Supplementary material for: A versatile active learning workflow for optimization of genetic and metabolic networks
Source: Nat Commun. 2022 Jul 5;13:3876. doi: 10.1038/s41467-022-31245-z (PMC9256728; doi:10.1038/s41467-022-31245-z)
Supplement: Supplementary file 1 — Supplementary Information [file 41467_2022_31245_MOESM1_ESM.pdf]

Supplementary information for

A versatile active learning workflow for optimization of genetic and metabolic networks

Amir Pandi[1,9]*, Christoph Diehl[1,9], Ali Yazdizadeh Kharrazi[2], Scott A. Scholz[1], Elizaveta Bobkova[1], Léon Faure[3], Maren Nattermann[1], David Adam[1], Nils Chapin[1], Yeganeh Foroughijabbari[1], Charles Moritz[1], Nicole Paczia[4], Niña Socorro Cortina[1,5], Jean-Loup Faulon[3,6,7], and Tobias J. Erb*[1,8]

[1] Department of Biochemistry & Synthetic Metabolism, Max Planck Institute for Terrestrial Microbiology, Marburg, Germany
[2] DataChef, Amsterdam, The Netherlands
[3] Micalis Institute, INRAE, AgroParisTech, University of Paris-Saclay, Jouy-en-Josas, France
[4] Core Facility for Metabolomics and Small Molecule Mass Spectrometry, Max Planck Institute for Terrestrial Microbiology, Marburg, Germany
[5] LiVeritas Biosciences, Inc., 432 N Canal St.; Ste. 20, South San Francisco CA 94080, USA
[6] Genomique Metabolique, Genoscope, Institut Francois Jacob, CEA, CNRS, Univ Evry, University of Paris-Saclay, Evry, France
[7] Manchester Institute of Biotechnology, SYNBIOCHEM center, School of Chemistry, The University of Manchester, Manchester, UK
[8] SYNMIKRO Center of Synthetic Microbiology, Marburg, Germany
[9] Amir Pandi and Christoph Diehl contributed equally to this work

* To whom correspondence should be addressed: toerb@mpi-marburg.mpg.de and amir.pandi@mpi-marburg.mpg.de

The supplementary information contains supplementary notes, tables, figures, and references:

Supplementary Notes 1-7 (p. 3-9)

Supplementary Note 1: Active learning process for optimization of cell-free Gfp production.

Supplementary Note 2: User guide and description of features of the modular workflow.

Supplementary Note 3: Complementary note on the experiment and discussion for optimization of *LacI* gene circuits.

Supplementary Note 4: Setup for the METIS based optimization of the CETCH cycle.

Supplementary Note 5: Optimization versus prediction purpose.

Supplementary Note 6: Application of METIS prediction for simulation on a PURE cell-free system dataset.

Supplementary Note 7: Application of the workflow for combinatorial enzyme engineering.

Supplementary Tables 1-9 (p. 10-18)

Supplementary Table 1: Summary of METIS optimization (also known as Bayesian optimization) and prediction Google Colab packages.

Supplementary Table 2: The removed conditions after the first 3 rounds of yield active learning.

Supplementary Table 3: Timepoint assays of 7 selected conditions with their active learning yield (AL yield) and efficiency (AL efficiency) values.

Supplementary Table 4: Gradient for the separation of CoA esters.

Supplementary Table 5: Multiple reaction monitoring (MRM) transitions for measurement of CoA esters.

Supplementary Table 6: Gradient for the separation of glycolate.

Supplementary Table 7: Multiple reaction monitoring (MRM) transitions for measurement of glycolate.

Supplementary Table 8: Primer pairs used to PCR the *sfGfp* gene in Fig. 4.

Supplementary Table 9: Primer sequences used to PCR the *sfGfp* gene in Fig. 4

Supplementary Figures 1-22 (p. 19-38)

Supplementary Fig. 1: Comparison of performance between linear regressors and XGBoost algorithms with different number of data points in rounds of active learning.

Supplementary Fig. 2: Standard curve for fluorescence readout of purified sfGfp at the condition of the reference cell-free reaction.

Supplementary Fig. 3: Concentration variations of each of the *E. coli* cell-free system factors from rounds (days) 1 to 10 of active learning, and mutual interactions between every two factors for active learning of the *E. coli* cell-free system.

Supplementary Fig. 4: A screenshot of the Google Colab notebook for interacting with the workflow.

Supplementary Fig. 5: A screenshot of the sections of active learning parameters and factors.

Supplementary Fig. 6: A screenshot of different modules of the workflow.

Supplementary Fig. 7: The behavior of *LacI* gene circuits in the cell-free protein expression system.

Supplementary Fig. 8: Variation of the concentration of factors in 10 rounds of active learning for *LacI* gene circuits optimization.

Supplementary Fig. 9: Gfp fluorescence level is affected by expression competition with *LacI*, not by interference by the LacI protein itself.

Supplementary Fig. 10: Analysis of the active learning data for the transcription and translation unit.

Supplementary Fig. 11: Cell-free versus *in vivo* yields of the 20 most informative combinations for the transcription & translation unit.

Supplementary Fig. 12: Scaled factors heatmap of top 10% yields in CETCH yield (glycolate, round 1-5) active learning.

Supplementary Fig. 13: Scaled factors heatmap of top 10% yields in CETCH efficiency (glycolate concentration divided by the total enzyme concentration, round 1-8) active learning.

Supplementary Fig. 14: Distribution of measured yield (glycolate, round 1-5) values within the ranges of each factor.

Supplementary Fig 15: Distribution of measured efficiency (glycolate concentration divided by the total enzyme concentration, round 1-8) values within the ranges of each factor.

Supplementary Fig. 16: Assays for testing newly purified enzyme batches after round 2 and for testing different concentrations of hbs and cobalt.

Supplementary Fig. 17: Quantified CoA-esters from selected assay conditions.

Supplementary Fig. 18: Application of METIS for the prediction of a PURE cell-free system dataset.

Supplementary Fig. 19: Simulations on a dataset of 847 mutants of oxalyl-CoA decarboxylase enzyme to showcase the application of METIS workflow for combinatorial enzyme engineering.

Supplementary Fig. 20: Distribution of amino acids in each position of the oxalyl-CoA decarboxylase active site predicted by the model out of 50 suggestions.

Supplementary Fig. 21: DART vs default (gbtree) XGBoost algorithms and SMOGN data preprocessing.

Supplementary Fig. 22: Batch Upper Confidence Bound (UCB) for scoring objective functions.

Supplementary References (p. 39)

Supplementary Notes:

Supplementary Note 1: Active learning process for optimization of cell-free Gfp production
We started the active learning cycle by generating 20 random compositions for the first round, pipetting them, measuring the Gfp fluorescence in a plate reader, then we collected the results after 6 hours of incubation at 30 °C. The mean and standard deviation of each composition was imported to the model, and after training, it suggested a new set of 20 compositions. The yield was calculated from the measured Gfp fluorescence of every composition normalized by a composition in which all variable factors are at their mid-range. This normalization was used for active learning (**Data availability**). In the end, as plotted in **Fig. 1e**, all values were normalized by composition from a common protocol in the field of cell-free synthetic biology to be comparable with other studies (**Methods**). The cycle (**Fig. 1d**) was repeated for 10 rounds. In each round, the model became more predictive in generating new compositions and ranking the best 20 suggestions for the next round. Since pipetting 13 elements in 20 samples is error-prone and needs a substantial amount of time and effort, we developed a table-to-speech virtual assistant that is run on Google Colab and can be easily used on a computer or smartphone. This tool takes as input the suggested table of volumes to pipette, goes through it line by line (factors), ranks them from minimum to maximum volume, and reads and graphically shows them on the screen. With organized pipette tip sets and destination tubes (**Methods**), this considerably improved the speed, accuracy, and comfort of pipetting of such complex compositions.

Supplementary Note 2: User guide and description of features of the modular workflow
Fig. 2b shows the workflow of using METIS from the adjustment of the parameters to data visualization and analysis. The user input consists of two sections for i) active learning parameters and ii) factors with a range and/or category. In the active learning parameters section, the user should define the number of combinations willing to perform in each round depending on the number of factors and their conditions, also considering the equipment for i) pipetting compositions (or cloning genetic constructs in case of biological sequences) and ii) measuring the objective function. The number of rounds of active learning also should be defined as any arbitrary number. It is important to note that the number of total experiments is recommended to be divided into more rounds as this improves the power of active learning[1]. Other parameters are the total volume of each composition, the portion of the volume that could be varied excluding and listing the constant factors. This is important to specify the total volume and the volume of fixed components subtracted from it because the model needs to convert concentrations to volumes and vice versa. The minimum droplet size defines the minimum volume unit of liquid handling robots or the minimum unit that one can pipette, usually 0.2 or 0.5 µL. This means all the volumes proposed by the model will be a factor of 0.2/0.5 µL or, for example, 2.5/25 nL with Echo® acoustic robot.

The exploration/exploitation ratio referred to as "exploration" in the parameters section is one of the most important elements to thoughtfully define, especially when the user applies the workflow for a different application than those presented in this work. For applications with numerical factors (i.e., our examples in **Fig. 1, 3**), the exploration rate should be >1 for the early rounds of active learning, and toward the end of the cycle, it should tend to values <1. Importantly, these values depend on the number, importance, and type (numerical or categorical) of factors, therefore, for tailoring the workflow, it should be taken into account. A high exploration indicates that the model will take more risks in suggesting combinations (exploration) rather than being efficient (exploitation). High exploration ratios are needed in the early rounds to explore the space and escape from local optimal combinations. However, in the later rounds, more efficient suggestions are required, which corresponds to focusing on achieving higher yields using what the model has learned. The ratio should be very carefully assigned, and the users should rely on their knowledge of the system or check a few of the suggestions, especially for the use with categorical factors like for our examples in **Fig. 4, Supplementary Fig. 19**. Note that, even if an exploration ratio is assigned for Day 1 (in the absence of Day 0), the first step is fully randomized, and the ratio has no effect on it. The

exploration ratio for the examples reported in this work is as follows. 10 rounds of Gfp production in the cell-free system (**Fig. 1e**): Not defined, 1.41, 1, 1, 1, 1, 0.5, 0.5, 0.5, for 10 rounds of *LacI* gene circuit optimization (**Fig. 3c**): Not defined, 1.41, 1.0, 1.0, 1.41, 1.41, 1.41, 1.41, 1.0, 0.5, for 2 rounds of 20 most informative combinations of *LacI* gene circuits with purified LacI (**Fig. 3i**): Not defined for Day 0, 0.5 for Day 1, for 4 rounds for the transcription and translation unit (**Fig. 4c**): Not defined, 1.41, 4.0, 1.0, for 2 rounds of 20 most informative combinations of transcription translation unit *in vivo* (**Fig. 4f**): Not defined for Day 0, 2 for Day 1, and for the single round of enzyme engineering suggestions for the hypothetical next day (**Supplementary Fig. 20**): 2.0. It should be noted that, since in METIS optimization combinations are sorted based on only their std value and exploitation is set to zero (this enables picking the most uncertain combination for the next round (**Methods**)), the exploration ratio is not defined.

In the factors section (**Fig. 2b**), the user should list all the elements participating in the objective function, that can be categorical and/or numerical features. Numerical features are defined by values, such as the concentration of factors in an *in vitro* system or in a growth medium, or strength of regulatory sequences such as promoters, RBSs (ribosome binding site), or CRISPR RNAs. Categorical features are not defined by values but characters/names such as regulatory sequences without numerical scores, or when for a gene there are multiple candidates from different organisms. Combined features are those with categories (alternative sequences/genes/constructs) and their level through concentration or strength (*LacI* gene circuits in **Fig. 3**). The user can range numerical values either by giving the range boundaries (minimum and maximum) or by specifying all the values. After running this section, for factors with range boundaries, it might be the case that the number of conditions is high, or their distribution lacks the desired coverage within the range. In this situation, we suggest picking 5 to 10 values from the proposed conditions from the output code of this section and manually specifying those factors' concentrations. The stocks' concentration is an important parameter since it must support the volume of the minimum final concentration in the defined range dependent on the minimum volume unit. One highlighted feature of our workflow that empowers its performance is letting the range of factors have more variable values than only 4 conditions in Borkowski *et al*[2]. The more randomized space allows the model to sample more informatively within the ranges, and this flexibility improves the performance. After the user has defined the active learning parameters and factors, the model shows all ranges taking into account the final volume of the mixture to give a percentage of possible compositions. A percentage of 100 means it is possible to compose a condition in which all factors are at their highest volume (concentration) and lower percentages indicate compositions are limited. When needed, the stocks' concentration can be changed, and if on the other hand it limits the lowest volume, two stocks with different concentrations can be provided. This is one of the features of the workflow that can solve issues when it comes to practice.

If a pre-existing dataset is used to train the model prior to starting active learning, they can be imported (compositions and yields) to initiate the workflow on Day 0 instead of Day 1 (used examples for 20 most informative combinations in **Fig. 3i, 4f** and the enzyme engineering application in **Supplementary Note 7, Supplementary Fig. 19, 20**). The dataset should be provided in the format of the results.csv files (**Data availability**).

When the parameters and factors are all set up, the model can generate the first round of experiments from the file Volumes_1.csv (round 1). The model generates Volumes.csv and Concentration.csv files in each round. The user should perform the suggested experiments, measure the objective function, and insert the results (mean and standard deviations) into new columns in the Concentrations_1.csv file generated by the model for that round and rename it to Results_1.csv. To continue with the next day (round), the user should upload the Results_1.csv file into the Google Colab notebook, run all sections, and the model executes the next day's compositions. In this step, the model trains itself on the results and suggests new compositions predicted to give higher values for the objective function (or more predictive combinations in METIS prediction to build a more predictive model and not necessarily optimizing the objective function). This cycle is repeated for n that is defined in the parameters section. The file used for pipetting is Volume.csv

generated in each round. If the laboratory accesses Echo® acoustic liquid handler, our workflow can generate an input file corresponding to the suggested compositions. In this case, the source and destination plate wells should be specified by the user after downloading the Echo® file. Otherwise, compositions can be pipetted by other liquid handlers or manually using the Volume.csv file. In examples in **Fig. 1, 3**, for 20 compositions in each round, it took us around three hours to pipette all compositions using our table-to-speech virtual assistant. The table-to-speech assistant increases the speed, accuracy, and ease of pipetting that should be used through a separate Google Colab notebook that we also provided in this work. The notebook takes the Table2Speech_Volume.csv file as input, ranks, and reads them throughout all compositions for each factor. By adjusting the parameters in Google Colab the assistant will go to the next well with any click specified by the user (**Supplementary Note 1, Code availability**). If the system is as in our examples in **Fig. 4, Supplementary Fig. 20**, the combinations should be constructed or cloned, the Volume.csv lists the categories of factors that should be constructed and not real volumes to pipette.

During active learning, parameters or ranges can be altered at any round. This feature enables the user to make readjustments for the next rounds depending on the model's performance, the evolution of the objective function over rounds, and how factors behave within their ranges. Additionally, in the section named "specials", customized compositions can be manually added to any round and these will be subtracted from the number of suggestions in that round. If a user is willing to readjust or import special compositions, we recommend doing this in the later rounds of active learning and let the model perform its task in early rounds. For the *LacI* circuit optimization cycle, we show examples where customized compositions have been manually added. Controls (i.e., negative control or reference combination) could be part of specials, in particular when an Echo® file is used.

The workflow generates cumulative outputs in each round (see **Methods** for how they are mathematically/statistically generated). The first set of outputs are compositions as Concentrations.csv and Volumes.csv. The second set is the list of K most informative combinations. This list can be used after active learning, to facilitate optimization of similar systems with the same type of factors and objective function such that one does not need to redo the whole active learning. By doing experiments for K (user-defined) combinations and measuring their objective function, results are imported as Day 0 to optionally continue with one or more rounds of active learning (see *LacI* circuit section). The last set of outputs are several types of analysis/visualization and the respective raw data, a box plot for the evolution of objective function over rounds, two groups of individual plots for each factor one showing the daily variation of factors within their ranges, and the other group is about all measured yields within ranges. The other analysis/visualization outputs are a plot and list of feature importance values, contribution of each factor in the prediction of yield by the model. These provide information on factors playing more important roles and which have less or no effect on the objective function. The last module is a heatmap of mutual interactions between every two factors providing useful results to optimize and study the system as well as to spotlight unknown interactions.

Supplementary Note 3: Complementary note on the experiment and discussion for optimization of *LacI* gene circuits

The median of objective function increased especially from Day 6, and the fold-change raised from less than 4 to over 8 on Day 10 (**Fig. 3c**). The median on Day 9 dropped because we included 5 of 20 as special compositions with high concentrations of P_{T7}-*LacI* plasmid and purified T7 RNA polymerase that led to very low objective function values. We did so since the model was trending to suggest very low concentrations of these two factors (**Supplementary Fig. 8**) which we first assumed would improve the fold change and objective function. Although this manipulation in the active learning process reduced the objective function values on Day 9, we gained a more profound insight into the system's behavior. Additionally, this helped the model to improve the objective function values at the highest on Day 10. We hypothesize that the low protein production is because of resource competition or inhibitory protein-protein

interactions especially at high concentrations of the plasmid that higher amounts of amino acids and tRNAs could not compensate for it. This was noticed by the model such that during the 10-day period it was tending to suggest lower concentrations of P_{T7}-*LacI* plasmid and T7 RNA polymerase (**Fig. 3d, Supplementary Fig. 8**). **Fig. 3d, e** represent yield data points within each factor's range and the features' importance, respectively. The model calculated that P_{T7}-*LacI* plasmid is by far the most important feature because of its hugely negative effect on protein production (**Fig. 3e**).

To test the hypothesis of resource competition or inhibitory protein-protein interaction, we designed titration experiments in which with the optimal composition from active learning (which was with the pTHS circuit) we varied the concentration of P_{T7}-*LacI* plasmid and T7 RNA polymerase (**Fig. 3f**). Increasing the concentration of P_{T7}-*LacI* plasmid or T7 RNA polymerase diminishes the fold-change $\times$ dynamic range (FC $\times$ DR) and fold change (FC). The maximum of these values was achieved at low concentrations of P_{T7}-*LacI* plasmid and the highest amount of T7 RNA polymerase. To further support this hypothesis, instead of pTHS circuit, we added a *Gfp* expressing plasmid with a constitutive promoter that has no interaction with the P_{T7}-*LacI* plasmid and T7 RNA polymerase but shares the same resources for transcription and translation. Increasing the concentration of either P_{T7}-*LacI* or T7 RNA polymerase depleted resources for expression of the constitutive *Gfp* (**Fig. 3g**). Since the addition of the second plasmid prevented reaching a sufficient production-repression balance, we sought to test the system with His-tag purified LacI. We extracted the 20 most informative combinations from the above active learning cycle. We removed T7 RNA polymerase from the factors and replaced the P_{T7}-*LacI* plasmid with His-tag purified LacI and gave random values to it (**Fig. 3h**). These 20 combinations were performed, the results were collected and imported as Day 0 to the workflow. We already could see a huge improvement in the objective function and fold-change on Day 0 (**Fig. 3i**) due to using purified LacI. As high yield values were associated with the lower concentrations of purified LacI, we widened the range of purified LacI toward lower concentrations for the next round (Day 1). The Day 1 experiment resulted in 4 data points with a fold change of around 100 (right plot in **Fig. 3i**).

Supplementary Note 4: <u>Setup for the METIS based optimization of the CETCH cycle</u>
For the optimization of the CETCH cycle, we did 125 conditions in triplicates per round. In each condition, all components were variable except for the substrate propionyl-CoA, which was fixed to 100 µM. For the buffer (Hepes), we not only wanted to optimize the concentration, but also test different pH. Therefore, we gave the possibility to choose between 6 different pHs (7.0, 7.2, 7.4, 7.6, 7.8 and 8.0). For the five rounds of unrestricted optimization, we used exploration values of 1.41, 1.41, 1.0, 1.0 and 0.5. After transforming the data for the efficiency optimization ([Glycolate]/[Enz_{tot}]= Gycolate yield in µM and total concentration of enzymes in µM), we did three additional rounds of efficiency optimization where we used exploration values of 1.0, 0.5 and 0.5.

After the first two rounds, we purified new batches of mco, hbd, cat and ssr. In the third round, using the new enzyme stocks, we noticed that our positive control (see material and methods) had a roughly three times higher product yield compared to the first two rounds. Therefore, we tested our control manually with the new enzymes and 4 control setups where only the old enzyme stock of either mco, hbd, cat or ssr was used. We could identify the old catalase stock as the reason for the lower yields in the first two rounds (**Supplementary Fig. 16b**). We stored the new catalase stock in liquid nitrogen upon use. Additionally, we removed unnecessary values after the first three rounds to reduce the combinatorial space (see **Supplementary Table 2**). All other values are in the GitHub repository.

<u>Assays for determination of new enzyme stocks after round two (**Supplementary Fig. 16b**)</u>
The assays were done in triplicates containing 30 µL volume each and were carried out in a 1.5 mL reaction tube (at 30 °C, 500 rpm). The reactions were started with 100 µM propionyl-CoA. 8 µL samples were taken and quenched in 1 µL 50% formic acid and 1 µL 500 mM sodium polyphosphate (emplura®) at 1, 2, and 3 h. The samples were spun for 20 min at 4 °C and 20.000g, before the supernatant was transferred into new

tubes. As described earlier, the samples were diluted and mixed with the internal standard for LC-MS measurement.
The assays contained:
100 mM HEPES pH 7.6, 5 mM $MgCl_2$, 50 mM sodium bicarbonate, 20 mM sodium formate, 20 mM creatine phosphate, 0.5 mM CoA, 0.1 mM CoB_{12}, 2 mM ATP, 5 mM NADPH, 3.06 µM pco, 0.62 µM ccr, 0.74 µM epi, 0.30 µM mcm, 2.62 µM scr, 0.53 µM ssr, 5.34 µM hbs, 0.73 µM hbd, 0.58 µM ecm, 21.90 µM mco, 0.28 µM mch, 2.79 µM mcl, 1.64 µM cat, 14.56 fdh, 0.02 µM ca, 1.10 µM gor, 0.39 µM ck.
The experiments labeled mco, hbd, cat and ssr were done with the old stocks of the enzymes used in the first two rounds. The controls contained enzymes from the four new enzymes batches.

Assays for different concentrations of hbs and cobalt (**Supplementary Fig. 16a**)
The assays were done in triplicates containing 30 µL volume each and were carried out in a 1.5 mL reaction tube (at 30 °C, 500 rpm). The reactions were started with 100 µM propionyl-CoA. 8 µL samples were taken and quenched in 1 µL 50% formic acid and 1 µL 500 mM sodium polyphosphate (emplura®) at 1, 2 and 3 h. The samples were spun for 20 min at 4 °C and 20.000g, before the supernatant was transferred into new tubes. As described earlier, the samples were diluted and mixed with the internal standard for LC-MS measurement.

The hbs assays contained:
100 mM HEPES pH 7.6, 5 mM $MgCl_2$, 50 mM sodium bicarbonate, 20 mM creatine phosphate, 0.5 mM CoA, 0.1 mM CoB_{12}, 2 mM ATP, 10 mM NADPH, 10 mM NADH, 3.06 µM pco, 0.62 µM ccr, 0.74 µM epi, 0.30 µM mcm, 2.62 µM scr, 0.53 µM ssr, {0.53 µM hbs (10%), 5.34 µM hbs (control), 26.7 µM hbs (500%)}, 0.73 µM hbd, 0.58 µM ecm, 21.90 µM mco, 0.28 µM mch, 2.79 µM mcl, 1.64 µM cat, 0.02 µM ca, 1.10 µM gor, 0.39 µM ck.

The cobalt assays contained:
100 mM HEPES pH 7.6, 5 mM $MgCl_2$, 50 mM sodium bicarbonate, 20 mM sodium formate, 20 mM creatine phosphate, 0.5 mM CoA, {1.0 mM cobalt (1 mM), 0.0 mM cobalt (0 mM)}, 2 mM ATP, 5 mM NADPH, 3.06 µM pco, 0.62 µM ccr, 0.74 µM epi, 0.30 µM mcm, 2.62 µM scr, 0.53 µM ssr, 5.34 µM hbs, 0.73 µM hbd, 0.58 µM ecm, 21.90 µM mco, 0.28 µM mch, 2.79 µM mcl, 1.64 µM cat, 14.56 fdh, 0.02 µM ca, 1.10 µM gor, 0.39 µM ck.

Supplementary Note 5: Optimization versus prediction purpose
So far, we demonstrated the use of our active learning workflow for the experimentally guided optimization of cell-free protein expression, *LacI* gene circuit, transcription and translation unit, and the CETCH cycle. Machine learning algorithms learn patterns in data and predict unseen cases which can be either applied for optimization (as demonstrated with our workflow above) or prediction purposes. Note that while both approaches employ a dedicated prediction step, their overall aim is different (**Supplementary Table 1**). To further modularize our workflow, we created an additional METIS package for prediction (not optimization) of an objective function of given combinations. **Supplementary Table 1** summarizes the features of two Google Colab packages, METIS optimization and METIS prediction, which differ in goal and application, query strategy, and outputs. The query strategy or the approach of METIS optimization is maximizing the objective function as well as the standard deviation of the ensemble regressor (i.e., the result of multiple regressors that individually operate on the data) whereas METIS prediction only maximizes the standard deviation of the ensemble regressor. These two also differ in the output types; METIS optimization suggests combinations with high ranked objective function whereas METIS prediction generates a trained model that computes the objective function for input combinations.

Each round's suggestions in METIS prediction aim to build a more predictive model by maximizing the correlation between predicted and measured objective functions (and not necessarily maximizing the objective function as in METIS optimization), hence improve the R^2 of prediction over rounds of learning.

Beyond these differences, the two notebooks share the same features/modules as described in **Fig. 2** and **Supplementary Note 2**. To test the prediction notebook, we ran a simulation on a dataset of 1094 data points[3] from the PURE (purified recombinant elements) cell-free protein expression system in which recombinant proteins and the buffer composition were varied (**Supplementary Note 6, Supplementary Fig. xx**). To demonstrate the performance of the model, we split the dataset into train and validation sets and used the validation set to measure (using R^2) the accuracy of the prediction on the test set. Because of the random nature of splitting, the simulation was repeated 5 times. (**Supplementary Fig. 18**). Overall, the workflow was able to improve the prediction of the objective function over rounds of active learning.

Supplementary Note 6: Application of METIS prediction for simulation on a PURE cell-free system dataset
To show METIS's potential in predicting an objective function, we performed an active learning simulation on an available PURE (purified recombinant elements) cell-free system dataset[3] (**Supplementary Fig. 18a, b**). We also assessed the predictability of the dataset and our model's general performance, we calculated 5-fold cross-validation on the whole dataset (1098 data points). The result of 5-fold cross-validation and the prediction of a single sample test set is shown in **Supplementary Fig. 18c, d**, respectively.

We simulated an active learning cycle as presented in **Supplementary Fig. 18a**:

- 20% of the dataset was separated as the test set to be used for validation, which was not taken into account when the model is trained on the training set (the other 80%).
- In the first round, 80 data points from the training dataset were selected randomly and the model was trained on them.
- In the next round, using what the model has learned, the rest of the training set (80 subtracted) was sorted based on the uncertainty value of combinations (**Methods**) and the top 80 combinations were picked.
- The yield of the picked combinations was assigned from the dataset and the model was trained on them.
- This process was repeated 10 times, hence, at the end the model was trained on 800 data points.
- In each step performance of the model was evaluated on the test set.

This process includes multiple random steps which is why we repeated the whole process five times as shown in **Supplementary Fig 18a**.

Supplementary Note 7: Application of the workflow for combinatorial enzyme engineering
Machine learning is a powerful tool to address complicated biological questions such as prediction/engineering of the structure/activity of proteins/enzymes[4,5]. Challenges in engineering a protein using machine learning tools are the dependency on large datasets and difficulties to make genotype-phenotype links for hundreds or thousands of variants[4]. Except for phenotypes easy to measure such as those related to regulatory sequences (transcription factors), there is a lack of modular characterization methods for engineering proteins. Enzymes are of difficult proteins to engineer because each enzyme requires a different characterization method which mainly allows for low to medium throughput experiments. Moreover, traditional enzyme engineering approaches which mostly rely on altering one amino acid at a time, are likely to be trapped into the local optima of the enzyme activity[6]. Since our tool is able to work with minimal datasets and the gradient boosting algorithm can capture mutual interactions, it can also be used for engineering enzymes. From a recent study in our lab, we took a dataset of mutants of oxalyl-CoA decarboxylase from *Methylorubrum extorquens*[7] to run simulations and give an example of how to use the workflow for such applications. **Supplementary Fig. 19a** shows the active site of the enzyme and surrounding amino acid residues.

We imported the dataset comprising 847 combinations of mutations in the active site of the enzyme (**Data availability**). As provided in the modular workflow, we first extracted and plotted the feature importance

values (**Supplementary Fig. 19b**). E135G and Y497F were calculated as conditions with the highest effect on the yield. Although such conclusions could be made by experimental biochemists analyzing the mutants, our tool can quantify the effect of various amino acids at each position on the activity of the enzyme. The tailored workflow that we created for protein/enzyme engineering is provided with extra modules for the analysis and visualization of a mutant dataset. One of these provides 5-fold cross-validation on the whole dataset (**Supplementary Fig. 19c**). K-fold cross-validation assesses the predictivity of machine learning models on an independent dataset (**Methods**). An average of 0.69 (Pearson R^2) for 5-fold cross-validation was achieved on this dataset. The other module plots the performance of the model on a single test set, 20% of the whole data, after being trained on the other 80%. **Supplementary Fig. 19d** shows predicted values of the test set versus their measured values for the mutants dataset which supports an existing correlation between predicted and actual values. Our enzyme engineering notebook also enables continuing with active learning cycles from an imported dataset (**Supplementary Fig. 20**). One can also start from scratch to engineer an enzyme by following a procedure similar to the examples shown in this study.

Preparation of the dataset for enzyme mutants

Data for the iterative saturation mutagenesis of MeOXC was acquired as described previously[7]. Briefly, a three-enzyme cascade was employed to turn over formaldehyde and formyl-CoA to glyoxylate. The last step in the cascade formed hydrogen peroxide, which was used to convert Ampliflu Red to resorufin, allowing fluorometric detection. Mutants were evaluated by two parameters. First, the maximal rate of signal production was determined (parameter A), and second, the final amount of signal produced after 2 hours of runtime was measured (parameter B). The assigned objective function to each mutant is the product of parameters A and B. All values were normalized to the wildtype. If possible, the specific mutations were matched to the dataset. Where no sequencing data was available, the average signal was determined and assigned to the missing mutants.

Supplementary Tables:

Supplementary Table 1: Summary of METIS optimization (also known as Bayesian optimization) and prediction Google Colab packages. In this study, we provided two modular Google Colab notebooks that can be adjusted for different applications as shown in the study. These two notebooks differ in applications, query strategy, inputs and outputs.

Notebook	Application	Query Strategy	Input/start	Output
METIS optimization	-Optimization of an objective function, i.e., a composition, pathway, genetic construct/circuit	-Maximize the objective function as well as the standard deviation of ensemble regressor	-Existing data<br>OR<br>-Start with randomly generated compositions	-Compositions of categorical and/or quantitative factors that lead to higher yields
METIS prediction	-Prediction of an objective function, i.e., a composition, pathway, genetic construct/circuit	-Maximize only the standard deviation of ensemble regressor	-Existing data<br>OR<br>-Start with randomly generated compositions	-A trained model that predicts the objective function of given compositions

Supplementary Table 2: The removed conditions after the first 3 rounds of yield active learning.

Compounds	Removed values
HEPES	25
MgCl2	22.5, 25
Creatine P	80, 100
Bicarbonate	100
Formate	100
B12	0.6, 0.8, 1.0
pco	0.1914, 0.3827, 0.669725, 1.243775
hbs	1.06785, 2.1357, 3.737475, 6.941025
mco	1.36885, 2.7377, 4.10655, 5.4754, 6.84425, 9.58195, 13.6885, 19.1639
fdh	1.456, 2.912, 5.824, 10.192
gor	0.55235, 1.1047, 1.65705, 2.2094

Supplementary Table 3: Timepoint assays of 7 selected conditions with their active learning yield (AL yield) and efficiency (AL efficiency) values. Blue, day 4, condition 29, pH 7.8; orange, day 7, condition 15, pH 7.8; red, day 7, condition 76, pH 7.2; black, control, pH 7.6. green, day 8, condition 17, pH 7.2; lavender, day 5, condition 17, pH 7.4; burgundy, day 5, condition 55, pH 7.8.

	HEPES	MgCl2	CP	Bicarb.	Form.	CoA	B12	ATP	NADPH	pco	ccr	epi	mcm	scr	ssr	hbs	hbd	ecm	mco	mch	mcl	kat	fdh	ca	gor	ck	**Manual Yield (3h)**	**AL yield**	**AL Efficiency**
blue	75	12.5	5	10.0	40	0.5	0.1	10	3.75	2.3	2.2	1.5	1.5	7.0	4.4	0.5	1.5	2.9	46.5	0.3	14.7	1.6	13.1	0.0	4.4	2.7	2262.5	2869.6	26.8
orange	75	12.5	60	2.5	20	0.4	0.0	3	3.75	3.1	1.9	0.7	2.9	3.5	1.7	0.5	0.7	1.4	26.0	0.3	3.6	3.3	30.6	0.1	5.0	0.8	2126.8	2617.7	30.5
red	150	7.5	20	20.0	40	0.4	0.0	9	2.50	2.3	2.2	3.7	0.3	1.7	4.4	1.6	0.4	2.6	113.6	2.3	8.4	4.9	23.3	0.1	3.9	2.7	1872.5	2586.8	14.5
black	100	5.0	20	50.0	20	0.5	0.1	2	5.00	3.1	0.6	0.7	0.3	2.6	0.6	5.3	0.7	0.6	21.9	0.3	2.8	1.6	14.6	0.0	1.1	0.4	959.6	-	-
green	175	10.0	10	5.0	80	0.4	0.1	9	2.50	2.3	0.6	3.0	0.3	5.2	1.1	1.6	0.4	2.3	26.0	1.4	14.7	1.6	40.8	0.1	2.8	2.0	920.2	919.9	8.7
lavender	50	2.5	5	2.5	5	0.4	0.1	7	7.50	4.0	2.8	0.7	1.2	9.6	2.2	1.6	1.1	2.6	46.5	0.6	1.2	8.2	7.3	0.1	4.7	3.1	682.7	627.9	6.4
burgundy	50	17.5	10	5.0	20	4.0	0.1	5	5.00	3.1	1.5	3.7	0.9	13.1	4.4	12.3	1.8	2.9	34.2	2.0	1.6	4.9	23.3	0.0	3.6	0.8	296.6	354	3.1

Supplementary Table 4: Gradient for the separation of CoA esters.

**Time [min]**	**A [%]**	**B [%]**
0.0	100	0
0.5	100	0
6.0	96	4
10.0	77	23
11.0	20	80
12.0	20	80
12.1	100	0
15.0	100	0

Supplementary Table 5: Multiple reaction monitoring (MRM) transitions for measurement of CoA esters.

Compound	Precursor Ion	Product Ion	Dwell	Fragmentor	Collision Energy	Cell Accelerator Volt.	Polarity
Ethylmalonyl-CoA (Quantifier)	882.1	331.2	25	380	41	5	Positive
Ethylmalonyl-CoA (Qualifier)	882.1	428	25	380	29	5	Positive
Methylsuccinyl-CoA (Quantifier)	882	375.1	25	380	33	5	Positive
Methylsuccinyl-CoA (Qualifier)	882	428	25	380	29	5	Positive
Mesaconyl-CoA (Quantifier)	880.1	375.1	25	380	25	5	Positive
Mesaconyl-CoA (Qualifier)	880.1	428	25	380	35	5	Positive
Succinyl-CoA (Quantifier)	868.1	361.1	25	380	35	5	Positive
Succinyl-CoA (Qualifier)	868.1	428.1	25	380	35	5	Positive
Methylmalonyl-CoA (Quantifier)	868.1	317.1	25	380	41	5	Positive
Methylmalonyl-CoA (Qualifier)	868.1	428	25	380	31	5	Positive
4-hydroxybutyryl-CoA (Quantifier)	854.1	347.1	25	380	37	5	Positive
4-hydroxybutyryl-CoA (Qualifier)	854.1	428	25	380	30	5	Positive
Crotonyl-CoA (Quantifier)	836.1	329	25	380	33	5	Positive
Crotonyl-CoA (Qualifier)	836.1	428	25	380	26	5	Positive
Propionyl-CoA (Quantifier)	824.1	317.1	25	380	31	5	Positive
Propionyl-CoA (Qualifier)	824.1	428	25	380	28	5	Positive
B-methylmalyl-CoA (Quantifier)	898.1	391.1	25	380	39	5	Positive
B-methylmalyl-CoA (Qualifier)	898.1	428.1	25	380	33	5	Positive

Supplementary Table 6: Gradient for the separation of glycolate.

Time [min]	A [%]	B [%]
0	100	0
4	100	0
6	0	100
7	0	100
7.1	100	0
12	100	0

Supplementary Table 7: Multiple reaction monitoring (MRM) transitions for measurement of glycolate.

Compound	Precursor Ion	Product Ion	Dwell	Fragmentor	Collision Energy	Cell Accelerator Volt.	Polarity
^{12}C-Glycolate (Quantifier)	75	47	150	380	9	5	Negative
^{12}C-Glycolate (Qualifier)	75	75	150	380	0	5	Negative
^{13}C-Glycolate (Quantifier)	77	48	150	380	9	5	Negative
^{13}C-Glycolate (Qualifier)	77	77	150	380	0	5	Negative

Supplementary Table 8: Forward (Fw) and reverse (Rvs) primer pairs used to PCR the *sfGfp* gene for constructs (combinations) used in Fig. 4. The constructs number is associated with combinations available at METIS GitHub repository. Primer sequences are provided in Supplementary Table 9.

Construct (combination)	Day 1 cell-free		Day 2 cell-free		Day 3 cell-free		Day 4 cell-free		Day 1 *in vivo*	
	**Fw primer**	**Rvs primer**	**Fw primer**	**Rvs primer**	**Fw primer**	**Rvs primer**	**Fw primer**	**Rvs primer**	**Fw primer**	**Rvs primer**
1	oAP209	oAP208	oAP240	oAP194	oAP300	oAP198	oAP330	oAP202	oAP330	oAP189
2	oAP210	oAP205	oAP259	oAP194	oAP275	oAP198	oAP321	oAP202	oAP318	oAP189
3	oAP211	oAP208	oAP260	oAP194	oAP301	oAP198	oAP331	oAP202	oAP366	oAP193
4	oAP212	oAP206	oAP261	oAP194	oAP302	oAP198	oAP323	oAP190	oAP367	oAP208
5	oAP213	oAP197	oAP262	oAP194	oAP303	oAP198	oAP332	oAP202	oAP269	oAP189
6	oAP214	oAP203	oAP263	oAP194	oAP304	oAP204	oAP333	oAP202	oAP263	oAP192
7	oAP215	oAP203	oAP264	oAP194	oAP305	oAP198	oAP329	oAP202	oAP368	oAP189
8	oAP216	oAP199	oAP265	oAP194	oAP260	oAP191	oAP304	oAP206	oAP369	oAP205
9	oAP217	oAP200	oAP266	oAP194	oAP306	oAP190	oAP226	oAP202	oAP370	oAP196
10	oAP218	oAP191	oAP267	oAP194	oAP307	oAP198	oAP316	oAP206	oAP371	oAP201
11	oAP219	oAP205	oAP268	oAP194	oAP308	oAP198	oAP334	oAP190	oAP216	oAP198
12	oAP220	oAP198	oAP269	oAP194	oAP309	oAP202	oAP280	oAP202	oAP373	oAP201
13	oAP221	oAP201	oAP270	oAP194	oAP310	oAP198	oAP335	oAP202	oAP305	oAP189
14	oAP222	oAP194	oAP271	oAP194	oAP311	oAP198	oAP336	oAP202	oAP374	oAP194
15	oAP223	oAP204	oAP272	oAP194	oAP312	oAP203	oAP272	oAP202	oAP375	oAP190
16	oAP224	oAP197	oAP273	oAP194	oAP261	oAP197	oAP227	oAP202	oAP376	oAP189
17	oAP225	oAP198	oAP274	oAP194	oAP313	oAP203	oAP337	oAP202	oAP377	oAP202
18	oAP226	oAP194	oAP275	oAP194	oAP260	oAP206	oAP338	oAP198	oAP378	oAP208
19	oAP227	oAP200	oAP276	oAP194	oAP262	oAP192	oAP316	oAP203	oAP379	oAP189
20	oAP228	oAP191	oAP277	oAP194	oAP261	oAP193	oAP339	oAP202		
21	oAP229	oAP203	oAP278	oAP203	oAP314	oAP191	oAP340	oAP202		
22	oAP230	oAP201	oAP279	oAP194	oAP261	oAP192	oAP323	oAP207		
23	oAP231	oAP200	oAP280	oAP203	oAP315	oAP198	oAP341	oAP202		
24	oAP232	oAP194	oAP240	oAP198	oAP316	oAP197	oAP342	oAP202		
25	oAP233	oAP194	oAP281	oAP194	oAP262	oAP190	oAP343	oAP202		
26	oAP234	oAP196	oAP282	oAP203	oAP317	oAP198	oAP344	oAP190		
27	oAP235	oAP203	oAP283	oAP194	oAP318	oAP204	oAP330	oAP190		
28	oAP236	oAP189	oAP284	oAP194	oAP316	oAP208	oAP345	oAP202		
29	oAP237	oAP200	oAP285	oAP194	oAP318	oAP197	oAP248	oAP202		
30	oAP238	oAP193	oAP286	oAP203	oAP318	oAP202	oAP346	oAP202		
31	oAP239	oAP199	oAP287	oAP194	oAP319	oAP204	oAP318	oAP190		
32	oAP240	oAP190	oAP288	oAP203	oAP320	oAP202	oAP347	oAP202		
33	oAP241	oAP189	oAP289	oAP194	oAP321	oAP198	oAP348	oAP203		
34	oAP242	oAP196	oAP290	oAP203	oAP320	oAP199	oAP307	oAP202		
35	oAP243	oAP198	oAP291	oAP203	oAP319	oAP197	oAP237	oAP202		
36	oAP244	oAP193	oAP292	oAP194	oAP224	oAP198	oAP349	oAP190		
37	oAP245	oAP196	oAP293	oAP194	oAP319	oAP195	oAP319	oAP198		
38	oAP246	oAP198	oAP294	oAP194	oAP322	oAP203	oAP350	oAP202		
39	oAP247	oAP205	oAP295	oAP194	oAP323	oAP206	oAP351	oAP202		
40	oAP248	oAP207	oAP278	oAP198	oAP222	oAP198	oAP352	oAP202		
41	oAP249	oAP203	oAP240	oAP189	oAP259	oAP199	oAP323	oAP189		
42	oAP250	oAP189	oAP240	oAP199	oAP319	oAP199	oAP353	oAP202		
43	oAP251	oAP207	oAP240	oAP203	oAP313	oAP194	oAP354	oAP190		
44	oAP252	oAP203	oAP296	oAP203	oAP324	oAP203	oAP355	oAP202		
45	oAP253	oAP206	oAP297	oAP194	oAP325	oAP204	oAP238	oAP202		
46	oAP254	oAP196	oAP282	oAP198	oAP326	oAP201	oAP356	oAP206		
47	oAP255	oAP204	oAP298	oAP194	oAP327	oAP202	oAP357	oAP202		
48	oAP256	oAP201	oAP299	oAP194	oAP328	oAP201	oAP251	oAP202		
49	oAP257	oAP192	oAP240	oAP206	oAP329	oAP198	oAP327	oAP190		
50	oAP258	oAP191	oAP240	oAP192	oAP325	oAP202	oAP348	oAP195		

Supplementary Table 9: Primer sequences used to PCR the *sfGfp* gene (see primer pairs in Supplementary Table 8) for constructs (combinations) used in Fig. 4.

Primer ID	Primer sequence
oAP189	CTGGTCTCAAAGCTTACTCCTTCAGTTCATCCATACCATG
oAP190	CTGGTCTCAAAGCTTACTCCCGCAGTTCATCCATACCATG
oAP191	CTGGTCTCAAAGCTTACTCCCACAGTTCATCCATACCATG
oAP192	CTGGTCTCAAAGCTTACTCCGTCAGTTCATCCATACCATG
oAP193	CTGGTCTCAAAGCTTACTTCTTCAGTTCATCCATACCATG
oAP194	CTGGTCTCAAAGCTTACTTCCGCAGTTCATCCATACCATG
oAP195	CTGGTCTCAAAGCTTACTTCCACAGTTCATCCATACCATG
oAP196	CTGGTCTCAAAGCTTACTTCGTCAGTTCATCCATACCATG
oAP197	CTGGTCTCAAAGCTTACCGCTTCAGTTCATCCATACCATG
oAP198	CTGGTCTCAAAGCTTACCGCCGCAGTTCATCCATACCATG
oAP199	CTGGTCTCAAAGCTTACCGCCACAGTTCATCCATACCATG
oAP200	CTGGTCTCAAAGCTTACCGCGTCAGTTCATCCATACCATG
oAP201	CTGGTCTCAAAGCTTATAACTTCAGTTCATCCATACCATG
oAP202	CTGGTCTCAAAGCTTATAACCGCAGTTCATCCATACCATG
oAP203	CTGGTCTCAAAGCTTATAACCACAGTTCATCCATACCATG
oAP204	CTGGTCTCAAAGCTTATAACGTCAGTTCATCCATACCATG
oAP205	CTGGTCTCAAAGCTTAGGTCTTCAGTTCATCCATACCATG
oAP206	CTGGTCTCAAAGCTTAGGTCCGCAGTTCATCCATACCATG
oAP207	CTGGTCTCAAAGCTTAGGTCCACAGTTCATCCATACCATG
oAP208	CTGGTCTCAAAGCTTAGGTCGTCAGTTCATCCATACCATG
oAP209	CAGGTCTCAAGGATTAATACGACTCACTAGAGGTCTAGAGTCACACAGGAAAGTACTAGATGCGTCACAAGTATAAAGGCGAAGAGCTGTTC
oAP210	CAGGTCTCAAGGATTAATACGACTCACTTTAGGTCTAGAGTCACACAGGAAAGTACTAGATGCGTACAAGTATCAAAGGCGAAGAGCTGTTC
oAP211	CAGGTCTCAAGGATTAATACGACTCACTTTAGGTCTAGAGAAAGAGGGGAAATACTAGATGCGTCAAGTATCAAAAGGCGAAGAGCTGTTC
oAP212	CAGGTCTCAAGGATTAATACGACTCACTATAGGTCTAGAGTTCACACAGGAAACCTACTAGATGCGTAAGTATGGTAAAGGCGAAGAGCTGTTC
oAP213	CAGGTCTCAAGGATTATTACGACTCACTATAGGTCTAGAGAAAGAGGGGAAATACTAGATGCGTCACAAGAAGAAAGGCGAAGAGCTGTTC
oAP214	CAGGTCTCAAGGATTAATGCGACTCACTATAGGTCTAGAGTCACACAGGAAAGTACTAGATGCGTAAGTTTTCAAAAGGCGAAGAGCTGTTC
oAP215	CAGGTCTCAAGGATTAATACGACTCACTAGAGGTCTAGAGATTAAAGAGGAGAAATACTAGATGCGTGACCCAGAGAAAGGCGAAGAGCTGTTC
oAP216	CAGGTCTCAAGGATTAATACGACTCACTATAGGTCTAGAGATTAAAGAGGAGAATACTAGATGCGTAAGTATGGTAAAGGCGAAGAGCTGTTC
oAP217	CAGGTCTCAAGGATTATTACGACTCACTATAGGTCTAGAGTCACACAGGAAAGTACTAGATGCGTCACAAGTATAAAGGCGAAGAGCTGTTC
oAP218	CAGGTCTCAAGGATTAGTACGACTCACTATAGGTCTAGAGATTAAAGAGGAGAAATACTAGATGCGTAAGAAGAAGAAAGGCGAAGAGCTGTTC
oAP219	CAGGTCTCAAGGATTATTACGACTCACTATAGGTCTAGAGTCACACAGGAAAGTACTAGATGCGTAAGTATGGTAAAGGCGAAGAGCTGTTC
oAP220	CAGGTCTCAAGGATTATTACGACTCACTATAGGTCTAGAGAAAGAGGAGAAATACTAGATGCGTAAGAAGTATAAAGGCGAAGAGCTGTTC
oAP221	CAGGTCTCAAGGATTAATGCGACTCACTATAGGTCTAGAGTCACACAGGAAAGTACTAGATGCGTGACCCAGAGAAAGGCGAAGAGCTGTTC
oAP222	CAGGTCTCAAGGATTAATACGACTCACTTTAGGTCTAGAGAAAGAGGAGAAATACTAGATGCGTAAGTATCACAAAGGCGAAGAGCTGTTC
oAP223	CAGGTCTCAAGGATTAATACGACTCACTAGAGGTCTAGAGTTCACACAGGAAACCTACTAGATGCGTAAGAAGTATAAAGGCGAAGAGCTGTTC
oAP224	CAGGTCTCAAGGATTAATACGACTCACTATAGGTCTAGAGATTAAAGAGGAGAAATACTAGATGCGTGACCCAGAGAAAGGCGAAGAGCTGTTC
oAP225	CAGGTCTCAAGGATTAATACGACTCACTTTAGGTCTAGAGAAAGAGGGGAAATACTAGATGCGTAAGAAGAAGAAAGGCGAAGAGCTGTTC
oAP226	CAGGTCTCAAGGATTAATGCGACTCACTATAGGTCTAGAGATTAAAGAGGAGAAATACTAGATGCGTAAGTTTTCAAAAGGCGAAGAGCTGTTC
oAP227	CAGGTCTCAAGGATTAATGCGACTCACTATAGGTCTAGAGTCACACAGGAAAGTACTAGATGCGTATTGGTAAGAAAGGCGAAGAGCTGTTC
oAP228	CAGGTCTCAAGGATTATTACGACTCACTATAGGTCTAGAGAAAGAGGAGAAATACTAGATGCGTGACCCAGAGAAAGGCGAAGAGCTGTTC
oAP229	CAGGTCTCAAGGATTAATACGACTCACTAGAGGTCTAGAGATTAAAGAGGAGAAATACTAGATGCGTATTGGTAAGAAAGGCGAAGAGCTGTTC
oAP230	CAGGTCTCAAGGATTATTACGACTCACTATAGGTCTAGAGTTCACACAGGAAACCTACTAGATGCGTCAAGTATCAAAAGGCGAAGAGCTGTTC
oAP231	CAGGTCTCAAGGATTAATACGACTCACTAGAGGTCTAGAGATTAAAGAGGAGAAATACTAGATGCGTAAGTTTTCAAAAGGCGAAGAGCTGTTC
oAP232	CAGGTCTCAAGGATTAATGCGACTCACTATAGGTCTAGAGAAAGAGGAGAAATACTAGATGCGTACCGTTGGTAAAGGCGAAGAGCTGTTC
oAP233	CAGGTCTCAAGGATTATTACGACTCACTATAGGTCTAGAGATTAAAGAGGAGAAATACTAGATGCGTAAGTATCACAAAGGCGAAGAGCTGTTC
oAP234	CAGGTCTCAAGGATTAATGCGACTCACTATAGGTCTAGAGATTAAAGAGGAGAAATACTAGATGCGTACCGTTGGTAAAGGCGAAGAGCTGTTC
oAP235	CAGGTCTCAAGGATTATTACGACTCACTATAGGTCTAGAGAAAGAGGAGAAATACTAGATGCGTTATAAGCAAAAAGGCGAAGAGCTGTTC
oAP236	CAGGTCTCAAGGATTAATACGACTCACTTTAGGTCTAGAGATTAAAGAGGAGAATACTAGATGCGTCACAAGTATAAAGGCGAAGAGCTGTTC
oAP237	CAGGTCTCAAGGATTATTACGACTCACTATAGGTCTAGAGAAAGAGGGGAAATACTAGATGCGTTATAAGAAGAAAGGCGAAGAGCTGTTC
oAP238	CAGGTCTCAAGGATTAATACGACTCACTATAGGTCTAGAGAAAGAGGGGAAATACTAGATGCGTCACAAGTATAAAGGCGAAGAGCTGTTC
oAP239	CAGGTCTCAAGGATTAATACGACTCACTTTAGGTCTAGAGAAAGAGGAGAAATACTAGATGCGTACCGTTGGTAAAGGCGAAGAGCTGTTC
oAP240	CAGGTCTCAAGGATTAATACGACTCACTATAGGTCTAGAGATTAAAGAGGAGAAATACTAGATGCGTACAAGTATCAAAGGCGAAGAGCTGTTC
oAP241	CAGGTCTCAAGGATTAATACGACTCACTTTAGGTCTAGAGTCACACAGGAAAGTACTAGATGCGTTATAAGCAAAAAGGCGAAGAGCTGTTC
oAP242	CAGGTCTCAAGGATTATTACGACTCACTATAGGTCTAGAGTTCACACAGGAAACCTACTAGATGCGTTATAAGAAGAAAGGCGAAGAGCTGTTC
oAP243	CAGGTCTCAAGGATTAATACGACTCACTAGAGGTCTAGAGATTAAAGAGGAGAATACTAGATGCGTGACCCAGAGAAAGGCGAAGAGCTGTTC

oAP244	CAGGTCTCAAGGATTATTACGACTCACTATAGGTCTAGAGAAAGAGGAGAAATACTAGATGCGTAAGAAGTATAAAGGCGAAGAGCTGTTC
oAP245	CAGGTCTCAAGGATTAATACGACTCACTAGAGGTCTAGAGATTAAAGAGGAGAAATACTAGATGCGTCACAAGTATAAAGGCGAAGAGCTGTTC
oAP246	CAGGTCTCAAGGATTAATACGACTCACTAGAGGTCTAGAGTTCACACAGGAAACCTACTAGATGCGTCACAAGAAGAAAGGCGAAGAGCTGTTC
oAP247	CAGGTCTCAAGGATTAATACGACTCACTATAGGTCTAGAGTCACACAGGAAAGTACTAGATGCGTAAGAAGAAGAAAGGCGAAGAGCTGTTC
oAP248	CAGGTCTCAAGGATTAATACGACTCACTTTAGGTCTAGAGTCACACAGGAAAGTACTAGATGCGTTATAAGAAGAAAGGCGAAGAGCTGTTC
oAP249	CAGGTCTCAAGGATTAATACGACTCACTTTAGGTCTAGAGATTAAAGAGGAGAATACTAGATGCGTCACAAGAAGAAAGGCGAAGAGCTGTTC
oAP250	CAGGTCTCAAGGATTAATACGACTCACTTTAGGTCTAGAGATTAAAGAGGAGAAATACTAGATGCGTAAGAAGAAGAAAGGCGAAGAGCTGTTC
oAP251	CAGGTCTCAAGGATTAATGCGACTCACTATAGGTCTAGAGAAAGAGGGGAAATACTAGATGCGTAAGAAGAAGAAAGGCGAAGAGCTGTTC
oAP252	CAGGTCTCAAGGATTAATGCGACTCACTATAGGTCTAGAGAAAGAGGAGAAATACTAGATGCGTAAGTATTATAAAGGCGAAGAGCTGTTC
oAP253	CAGGTCTCAAGGATTAATACGACTCACTAGAGGTCTAGAGATTAAAGAGGAGAATACTAGATGCGTATTGGTAAGAAAGGCGAAGAGCTGTTC
oAP254	CAGGTCTCAAGGATTAGTACGACTCACTATAGGTCTAGAGAAAGAGGGGAAATACTAGATGCGTAAGTATCACAAAGGCGAAGAGCTGTTC
oAP255	CAGGTCTCAAGGATTAATACGACTCACTAGAGGTCTAGAGTTCACACAGGAAACCTACTAGATGCGTACAAGTATCAAAGGCGAAGAGCTGTTC
oAP256	CAGGTCTCAAGGATTAATACGACTCACTTTAGGTCTAGAGTCACACAGGAAAGTACTAGATGCGTGACCCAGAGAAAGGCGAAGAGCTGTTC
oAP257	CAGGTCTCAAGGATTAATACGACTCACTTTAGGTCTAGAGTCACACAGGAAAGTACTAGATGCGTTATAAGCAAAAAGGCGAAGAGCTGTTC
oAP258	CAGGTCTCAAGGATTAATACGACTCACTAGAGGTCTAGAGAAAGAGGAGAAATACTAGATGCGTTATAAGCAAAAAGGCGAAGAGCTGTTC
oAP259	CAGGTCTCAAGGATTAATACGACTCACTATAGGTCTAGAGATTAAAGAGGAGAAATACTAGATGCGTAAGAAGTATAAAGGCGAAGAGCTGTTC
oAP260	CAGGTCTCAAGGATTAATACGACTCACTATAGGTCTAGAGATTAAAGAGGAGAAATACTAGATGCGTAAGTATCACAAAGGCGAAGAGCTGTTC
oAP261	CAGGTCTCAAGGATTAATACGACTCACTATAGGTCTAGAGATTAAAGAGGAGAAATACTAGATGCGTTATAAGCAAAAAGGCGAAGAGCTGTTC
oAP262	CAGGTCTCAAGGATTAATACGACTCACTATAGGTCTAGAGATTAAAGAGGAGAAATACTAGATGCGTATTGGTAAGAAAGGCGAAGAGCTGTTC
oAP263	CAGGTCTCAAGGATTAATACGACTCACTATAGGTCTAGAGATTAAAGAGGAGAAATACTAGATGCGTTATAAGAAGAAAGGCGAAGAGCTGTTC
oAP264	CAGGTCTCAAGGATTAATACGACTCACTATAGGTCTAGAGATTAAAGAGGAGAAATACTAGATGCGTAAGTATTATAAAGGCGAAGAGCTGTTC
oAP265	CAGGTCTCAAGGATTAATACGACTCACTATAGGTCTAGAGATTAAAGAGGAGAAATACTAGATGCGTAAGTTTTCAAAAGGCGAAGAGCTGTTC
oAP266	CAGGTCTCAAGGATTAATACGACTCACTATAGGTCTAGAGATTAAAGAGGAGAAATACTAGATGCGTCAAGTATCAAAAGGCGAAGAGCTGTTC
oAP267	CAGGTCTCAAGGATTATTACGACTCACTATAGGTCTAGAGATTAAAGAGGAGAAATACTAGATGCGTACAAGTATCAAAGGCGAAGAGCTGTTC
oAP268	CAGGTCTCAAGGATTATTACGACTCACTATAGGTCTAGAGATTAAAGAGGAGAAATACTAGATGCGTATTGGTAAGAAAGGCGAAGAGCTGTTC
oAP269	CAGGTCTCAAGGATTATTACGACTCACTATAGGTCTAGAGATTAAAGAGGAGAAATACTAGATGCGTTATAAGCAAAAAGGCGAAGAGCTGTTC
oAP270	CAGGTCTCAAGGATTAATACGACTCACTATAGGTCTAGAGATTAAAGAGGAGAAATACTAGATGCGTAAGTATGGTAAAGGCGAAGAGCTGTTC
oAP271	CAGGTCTCAAGGATTATTACGACTCACTATAGGTCTAGAGATTAAAGAGGAGAAATACTAGATGCGTTATAAGAAGAAAGGCGAAGAGCTGTTC
oAP272	CAGGTCTCAAGGATTATTACGACTCACTATAGGTCTAGAGATTAAAGAGGAGAAATACTAGATGCGTAAGTATGGTAAAGGCGAAGAGCTGTTC
oAP273	CAGGTCTCAAGGATTATTACGACTCACTATAGGTCTAGAGATTAAAGAGGAGAAATACTAGATGCGTAAGTATTATAAAGGCGAAGAGCTGTTC
oAP274	CAGGTCTCAAGGATTATTACGACTCACTATAGGTCTAGAGATTAAAGAGGAGAAATACTAGATGCGTAAGTTTTCAAAAGGCGAAGAGCTGTTC
oAP275	CAGGTCTCAAGGATTATTACGACTCACTATAGGTCTAGAGATTAAAGAGGAGAAATACTAGATGCGTAAGAAGTATAAAGGCGAAGAGCTGTTC
oAP276	CAGGTCTCAAGGATTATTACGACTCACTATAGGTCTAGAGATTAAAGAGGAGAAATACTAGATGCGTCAAGTATCAAAAGGCGAAGAGCTGTTC
oAP277	CAGGTCTCAAGGATTAATGCGACTCACTATAGGTCTAGAGATTAAAGAGGAGAAATACTAGATGCGTACAAGTATCAAAGGCGAAGAGCTGTTC
oAP278	CAGGTCTCAAGGATTATTACGACTCACTATAGGTCTAGAGAAAGAGGAGAAATACTAGATGCGTACAAGTATCAAAGGCGAAGAGCTGTTC
oAP279	CAGGTCTCAAGGATTAATACGACTCACTATAGGTCTAGAGATTAAAGAGGAGAAATACTAGATGCGTAAGAAGAAGAAAGGCGAAGAGCTGTTC
oAP280	CAGGTCTCAAGGATTATTACGACTCACTATAGGTCTAGAGAAAGAGGAGAAATACTAGATGCGTATTGGTAAGAAAGGCGAAGAGCTGTTC
oAP281	CAGGTCTCAAGGATTAATGCGACTCACTATAGGTCTAGAGATTAAAGAGGAGAAATACTAGATGCGTATTGGTAAGAAAGGCGAAGAGCTGTTC
oAP282	CAGGTCTCAAGGATTATTACGACTCACTATAGGTCTAGAGAAAGAGGAGAAATACTAGATGCGTAAGTATCACAAAGGCGAAGAGCTGTTC
oAP283	CAGGTCTCAAGGATTAATACGACTCACTTTAGGTCTAGAGATTAAAGAGGAGAAATACTAGATGCGTACAAGTATCAAAGGCGAAGAGCTGTTC
oAP284	CAGGTCTCAAGGATTAATACGACTCACTTTAGGTCTAGAGATTAAAGAGGAGAAATACTAGATGCGTATTGGTAAGAAAGGCGAAGAGCTGTTC
oAP285	CAGGTCTCAAGGATTAATGCGACTCACTATAGGTCTAGAGATTAAAGAGGAGAAATACTAGATGCGTAAGTATCACAAAGGCGAAGAGCTGTTC
oAP286	CAGGTCTCAAGGATTATTACGACTCACTATAGGTCTAGAGAAAGAGGAGAAATACTAGATGCGTTATAAGAAGAAAGGCGAAGAGCTGTTC
oAP287	CAGGTCTCAAGGATTAATGCGACTCACTATAGGTCTAGAGATTAAAGAGGAGAAATACTAGATGCGTTATAAGCAAAAAGGCGAAGAGCTGTTC
oAP288	CAGGTCTCAAGGATTATTACGACTCACTATAGGTCTAGAGAAAGAGGAGAAATACTAGATGCGTAAGTATGGTAAAGGCGAAGAGCTGTTC
oAP289	CAGGTCTCAAGGATTAATGCGACTCACTATAGGTCTAGAGATTAAAGAGGAGAAATACTAGATGCGTTATAAGAAGAAAGGCGAAGAGCTGTTC
oAP290	CAGGTCTCAAGGATTATTACGACTCACTATAGGTCTAGAGAAAGAGGAGAAATACTAGATGCGTAAGTTTTCAAAAGGCGAAGAGCTGTTC
oAP291	CAGGTCTCAAGGATTATTACGACTCACTATAGGTCTAGAGAAAGAGGAGAAATACTAGATGCGTAAGTATTATAAAGGCGAAGAGCTGTTC
oAP292	CAGGTCTCAAGGATTAATGCGACTCACTATAGGTCTAGAGATTAAAGAGGAGAAATACTAGATGCGTAAGTATGGTAAAGGCGAAGAGCTGTTC
oAP293	CAGGTCTCAAGGATTAATACGACTCACTTTAGGTCTAGAGATTAAAGAGGAGAAATACTAGATGCGTAAGTATCACAAAGGCGAAGAGCTGTTC
oAP294	CAGGTCTCAAGGATTAATGCGACTCACTATAGGTCTAGAGATTAAAGAGGAGAAATACTAGATGCGTAAGAAGTATAAAGGCGAAGAGCTGTTC
oAP295	CAGGTCTCAAGGATTAATGCGACTCACTATAGGTCTAGAGATTAAAGAGGAGAAATACTAGATGCGTAAGTATTATAAAGGCGAAGAGCTGTTC
oAP296	CAGGTCTCAAGGATTATTACGACTCACTATAGGTCTAGAGAAAGAGGAGAAATACTAGATGCGTCAAGTATCAAAAGGCGAAGAGCTGTTC
oAP297	CAGGTCTCAAGGATTAATACGACTCACTATAGGTCTAGAGATTAAAGAGGAGAAATACTAGATGCGTCACAAGAAGAAAGGCGAAGAGCTGTTC
oAP298	CAGGTCTCAAGGATTAATACGACTCACTTTAGGTCTAGAGATTAAAGAGGAGAAATACTAGATGCGTTATAAGAAGAAAGGCGAAGAGCTGTTC
oAP299	CAGGTCTCAAGGATTAATACGACTCACTTTAGGTCTAGAGATTAAAGAGGAGAAATACTAGATGCGTTATAAGCAAAAAGGCGAAGAGCTGTTC
oAP300	CAGGTCTCAAGGATTAGTACGACTCACTATAGGTCTAGAGATTAAAGAGGAGAAATACTAGATGCGTAAGAAGTATAAAGGCGAAGAGCTGTTC
oAP301	CAGGTCTCAAGGATTAATACGACTCACTAGAGGTCTAGAGATTAAAGAGGAGAAATACTAGATGCGTAAGAAGTATAAAGGCGAAGAGCTGTTC
oAP302	CAGGTCTCAAGGATTAATACGACTCACTATAGGTCTAGAGAAAGAGGGGAAATACTAGATGCGTAAGTATCACAAAGGCGAAGAGCTGTTC
oAP303	CAGGTCTCAAGGATTAATACGACTCACTTTAGGTCTAGAGATTAAAGAGGAGAATACTAGATGCGTAAGTATCACAAAGGCGAAGAGCTGTTC

oAP304	CAGGTCTCAAGGATTAATACGACTCACTATAGGTCTAGAGATTAAAGAGGAGAATACTAGATGCGTAAGTATCACAAAGGCGAAGAGCTGTTC
oAP305	CAGGTCTCAAGGATTATTACGACTCACTATAGGTCTAGAGAAAGAGGGGAAATACTAGATGCGTAAGTATCACAAAGGCGAAGAGCTGTTC
oAP306	CAGGTCTCAAGGATTAATACGACTCACTATAGGTCTAGAGATTAAAGAGGAGAATACTAGATGCGTAAGTATCACAAAGGCGAAGAGCTGTTC
oAP307	CAGGTCTCAAGGATTAATACGACTCACTAGAGGTCTAGAGAAAGAGGGGAAATACTAGATGCGTAAGAAGTATAAAGGCGAAGAGCTGTTC
oAP308	CAGGTCTCAAGGATTAATACGACTCACTAGAGGTCTAGAGATTAAAGAGGAGAAATACTAGATGCGTAAGTATCACAAAGGCGAAGAGCTGTTC
oAP309	CAGGTCTCAAGGATTATTACGACTCACTATAGGTCTAGAGATTAAAGAGGAGAATACTAGATGCGTAAGTATCACAAAGGCGAAGAGCTGTTC
oAP310	CAGGTCTCAAGGATTAATACGACTCACTAGAGGTCTAGAGATTAAAGAGGAGAATACTAGATGCGTACCGTTGGTAAAGGCGAAGAGCTGTTC
oAP311	CAGGTCTCAAGGATTATTACGACTCACTATAGGTCTAGAGATTAAAGAGGAGAAATACTAGATGCGTCACAAGTATAAAGGCGAAGAGCTGTTC
oAP312	CAGGTCTCAAGGATTAGTACGACTCACTATAGGTCTAGAGATTAAAGAGGAGAAATACTAGATGCGTATTGGTAAGAAAGGCGAAGAGCTGTTC
oAP313	CAGGTCTCAAGGATTAGTACGACTCACTATAGGTCTAGAGATTAAAGAGGAGAAATACTAGATGCGTTATAAGCAAAAAGGCGAAGAGCTGTTC
oAP314	CAGGTCTCAAGGATTAATACGACTCACTATAGGTCTAGAGATTAAAGAGGAGAATACTAGATGCGTATTGGTAAGAAAGGCGAAGAGCTGTTC
oAP315	CAGGTCTCAAGGATTAATACGACTCACTTTAGGTCTAGAGAAAGAGGGGAAATACTAGATGCGTAAGAAGTATAAAGGCGAAGAGCTGTTC
oAP316	CAGGTCTCAAGGATTAATACGACTCACTATAGGTCTAGAGATTAAAGAGGAGAATACTAGATGCGTTATAAGCAAAAAGGCGAAGAGCTGTTC
oAP317	CAGGTCTCAAGGATTATTACGACTCACTATAGGTCTAGAGTTCACACAGGAAACCTACTAGATGCGTAAGAAGTATAAAGGCGAAGAGCTGTTC
oAP318	CAGGTCTCAAGGATTATTACGACTCACTATAGGTCTAGAGATTAAAGAGGAGAATACTAGATGCGTTATAAGAAGAAAGGCGAAGAGCTGTTC
oAP319	CAGGTCTCAAGGATTATTACGACTCACTATAGGTCTAGAGATTAAAGAGGAGAATACTAGATGCGTATTGGTAAGAAAGGCGAAGAGCTGTTC
oAP320	CAGGTCTCAAGGATTATTACGACTCACTATAGGTCTAGAGATTAAAGAGGAGAATACTAGATGCGTTATAAGCAAAAAGGCGAAGAGCTGTTC
oAP321	CAGGTCTCAAGGATTATTACGACTCACTATAGGTCTAGAGATTAAAGAGGAGAATACTAGATGCGTAAGAAGAAGAAAGGCGAAGAGCTGTTC
oAP322	CAGGTCTCAAGGATTAATACGACTCACTTTAGGTCTAGAGATTAAAGAGGAGAATACTAGATGCGTTATAAGCAAAAAGGCGAAGAGCTGTTC
oAP323	CAGGTCTCAAGGATTAATACGACTCACTATAGGTCTAGAGATTAAAGAGGAGAATACTAGATGCGTAAGAAGTATAAAGGCGAAGAGCTGTTC
oAP324	CAGGTCTCAAGGATTAATGCGACTCACTATAGGTCTAGAGAAAGAGGAGAAATACTAGATGCGTTATAAGCAAAAAGGCGAAGAGCTGTTC
oAP325	CAGGTCTCAAGGATTAATGCGACTCACTATAGGTCTAGAGATTAAAGAGGAGAATACTAGATGCGTAAGTATCACAAAGGCGAAGAGCTGTTC
oAP326	CAGGTCTCAAGGATTAGTACGACTCACTATAGGTCTAGAGATTAAAGAGGAGAAATACTAGATGCGTAAGTATCACAAAGGCGAAGAGCTGTTC
oAP327	CAGGTCTCAAGGATTAATACGACTCACTATAGGTCTAGAGATTAAAGAGGAGAATACTAGATGCGTACAAGTATCAAAGGCGAAGAGCTGTTC
oAP328	CAGGTCTCAAGGATTAGTACGACTCACTATAGGTCTAGAGATTAAAGAGGAGAATACTAGATGCGTAAGTATCACAAAGGCGAAGAGCTGTTC
oAP329	CAGGTCTCAAGGATTAATACGACTCACTAGAGGTCTAGAGATTAAAGAGGAGAATACTAGATGCGTAAGTATTATAAAGGCGAAGAGCTGTTC
oAP330	CAGGTCTCAAGGATTAATACGACTCACTATAGGTCTAGAGATTAAAGAGGAGAATACTAGATGCGTTATAAGAAGAAAGGCGAAGAGCTGTTC
oAP331	CAGGTCTCAAGGATTAATGCGACTCACTATAGGTCTAGAGATTAAAGAGGAGAATACTAGATGCGTAAGTTTTCAAAAGGCGAAGAGCTGTTC
oAP332	CAGGTCTCAAGGATTAGTACGACTCACTATAGGTCTAGAGATTAAAGAGGAGAATACTAGATGCGTTATAAGCAAAAAGGCGAAGAGCTGTTC
oAP333	CAGGTCTCAAGGATTAATGCGACTCACTATAGGTCTAGAGTCACACAGGAAAGTACTAGATGCGTTATAAGCAAAAAGGCGAAGAGCTGTTC
oAP334	CAGGTCTCAAGGATTAATACGACTCACTTTAGGTCTAGAGATTAAAGAGGAGAATACTAGATGCGTAAGAAGTATAAAGGCGAAGAGCTGTTC
oAP335	CAGGTCTCAAGGATTAATGCGACTCACTATAGGTCTAGAGAAAGAGGAGAAATACTAGATGCGTTATAAGAAGAAAGGCGAAGAGCTGTTC
oAP336	CAGGTCTCAAGGATTAATACGACTCACTAGAGGTCTAGAGATTAAAGAGGAGAATACTAGATGCGTTATAAGAAGAAAGGCGAAGAGCTGTTC
oAP337	CAGGTCTCAAGGATTAATGCGACTCACTATAGGTCTAGAGAAAGAGGGGAAATACTAGATGCGTAAGAAGTATAAAGGCGAAGAGCTGTTC
oAP338	CAGGTCTCAAGGATTAATGCGACTCACTATAGGTCTAGAGATTAAAGAGGAGAATACTAGATGCGTTATAAGCAAAAAGGCGAAGAGCTGTTC
oAP339	CAGGTCTCAAGGATTAATACGACTCACTTTAGGTCTAGAGATTAAAGAGGAGAAATACTAGATGCGTCAAGTATCAAAAGGCGAAGAGCTGTTC
oAP340	CAGGTCTCAAGGATTATTACGACTCACTATAGGTCTAGAGTCACACAGGAAAGTACTAGATGCGTAAGTATCACAAAGGCGAAGAGCTGTTC
oAP341	CAGGTCTCAAGGATTAATACGACTCACTTTAGGTCTAGAGAAAGAGGAGAAATACTAGATGCGTAAGTATTATAAAGGCGAAGAGCTGTTC
oAP342	CAGGTCTCAAGGATTAATACGACTCACTAGAGGTCTAGAGTCACACAGGAAAGTACTAGATGCGTAAGAAGTATAAAGGCGAAGAGCTGTTC
oAP343	CAGGTCTCAAGGATTAATACGACTCACTAGAGGTCTAGAGATTAAAGAGGAGAATACTAGATGCGTAAGTATGGTAAAGGCGAAGAGCTGTTC
oAP344	CAGGTCTCAAGGATTAATACGACTCACTATAGGTCTAGAGAAAGAGGAGAAATACTAGATGCGTAAGAAGTATAAAGGCGAAGAGCTGTTC
oAP345	CAGGTCTCAAGGATTAATGCGACTCACTATAGGTCTAGAGTCACACAGGAAAGTACTAGATGCGTACAAGTATCAAAGGCGAAGAGCTGTTC
oAP346	CAGGTCTCAAGGATTAATACGACTCACTAGAGGTCTAGAGAAAGAGGAGAAATACTAGATGCGTAAGTATCACAAAGGCGAAGAGCTGTTC
oAP347	CAGGTCTCAAGGATTATTACGACTCACTATAGGTCTAGAGATTAAAGAGGAGAAATACTAGATGCGTACCGTTGGTAAAGGCGAAGAGCTGTTC
oAP348	CAGGTCTCAAGGATTAATGCGACTCACTATAGGTCTAGAGATTAAAGAGGAGAATACTAGATGCGTAAGAAGTATAAAGGCGAAGAGCTGTTC
oAP349	CAGGTCTCAAGGATTAATGCGACTCACTATAGGTCTAGAGATTAAAGAGGAGAATACTAGATGCGTTATAAGAAGAAAGGCGAAGAGCTGTTC
oAP350	CAGGTCTCAAGGATTAATACGACTCACTAGAGGTCTAGAGAAAGAGGAGAAATACTAGATGCGTAAGAAGAAGAAAGGCGAAGAGCTGTTC
oAP351	CAGGTCTCAAGGATTAATGCGACTCACTATAGGTCTAGAGTTCACACAGGAAACCTACTAGATGCGTAAGAAGTATAAAGGCGAAGAGCTGTTC
oAP352	CAGGTCTCAAGGATTAATGCGACTCACTATAGGTCTAGAGAAAGAGGGGAAATACTAGATGCGTAAGTATTATAAAGGCGAAGAGCTGTTC
oAP353	CAGGTCTCAAGGATTAATACGACTCACTAGAGGTCTAGAGAAAGAGGAGAAATACTAGATGCGTAAGTTTTCAAAAGGCGAAGAGCTGTTC
oAP354	CAGGTCTCAAGGATTAATACGACTCACTTTAGGTCTAGAGATTAAAGAGGAGAATACTAGATGCGTAAGTTTTCAAAAGGCGAAGAGCTGTTC
oAP355	CAGGTCTCAAGGATTAATACGACTCACTTTAGGTCTAGAGAAAGAGGGGAAATACTAGATGCGTTATAAGAAGAAAGGCGAAGAGCTGTTC
oAP356	CAGGTCTCAAGGATTAATACGACTCACTTTAGGTCTAGAGATTAAAGAGGAGAATACTAGATGCGTATTGGTAAGAAAGGCGAAGAGCTGTTC
oAP357	CAGGTCTCAAGGATTAATGCGACTCACTATAGGTCTAGAGTTCACACAGGAAACCTACTAGATGCGTATTGGTAAGAAAGGCGAAGAGCTGTTC
oAP366	CAGGTCTCAAGGATTAATACGACTCACTATAGGTCTAGAGTCACACAGGAAAGTACTAGATGCGTAAGTATTATAAAGGCGAAGAGCTGTTC
oAP367	CAGGTCTCAAGGATTAATACGACTCACTATAGGTCTAGAGAAAGAGGAGAAATACTAGATGCGTACAAGTATCAAAGGCGAAGAGCTGTTC
oAP368	CAGGTCTCAAGGATTATTACGACTCACTATAGGTCTAGAGATTAAAGAGGAGAAATACTAGATGCGTGACCCAGAGAAAGGCGAAGAGCTGTTC
oAP369	CAGGTCTCAAGGATTAATACGACTCACTATAGGTCTAGAGATTAAAGAGGAGAAATACTAGATGCGTCACAAGTATAAAGGCGAAGAGCTGTTC
oAP370	CAGGTCTCAAGGATTAATACGACTCACTATAGGTCTAGAGAAAGAGGAGAAATACTAGATGCGTAAGTATGGTAAAGGCGAAGAGCTGTTC
oAP371	CAGGTCTCAAGGATTAATACGACTCACTATAGGTCTAGAGTCACACAGGAAAGTACTAGATGCGTCACAAGTATAAAGGCGAAGAGCTGTTC

oAP372	CAGGTCTCAAGGATTAATACGACTCACTATAGGTCTAGAGTTCACACAGGAAACCTACTAGATGCGTTATAAGCAAAAAGGCGAAGAGCTGTTC
oAP373	CAGGTCTCAAGGATTAATACGACTCACTATAGGTCTAGAGAAAGAGGAGAAATACTAGATGCGTAAGAAGAAGAAAGGCGAAGAGCTGTTC
oAP374	CAGGTCTCAAGGATTAATACGACTCACTATAGGTCTAGAGTCACACAGGAAAGTACTAGATGCGTTATAAGAAGAAAGGCGAAGAGCTGTTC
oAP375	CAGGTCTCAAGGATTAATACGACTCACTATAGGTCTAGAGTTCACACAGGAAACCTACTAGATGCGTCACAAGAAGAAAGGCGAAGAGCTGTTC
oAP376	CAGGTCTCAAGGATTAATACGACTCACTTTAGGTCTAGAGAAAGAGGAGAAATACTAGATGCGTAAGAAGAAGAAAGGCGAAGAGCTGTTC
oAP377	CAGGTCTCAAGGATTAATACGACTCACTATAGGTCTAGAGAAAGAGGAGAAATACTAGATGCGTAAGTTTTCAAAAGGCGAAGAGCTGTTC
oAP378	CAGGTCTCAAGGATTAATACGACTCACTATAGGTCTAGAGTTCACACAGGAAACCTACTAGATGCGTATTGGTAAGAAAGGCGAAGAGCTGTTC
oAP379	CAGGTCTCAAGGATTAATGCGACTCACTATAGGTCTAGAGATTAAAGAGGAGAATACTAGATGCGTCACAAGTATAAAGGCGAAGAGCTGTTC

Supplementary Figures:

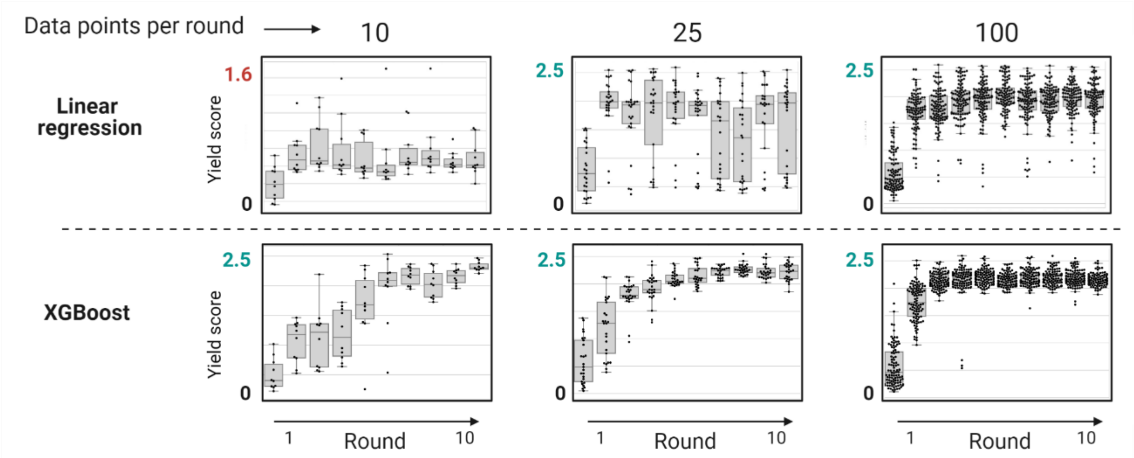

Supplementary Fig. 1: Comparison of performance between linear regressors and XGBoost algorithms with different number of data points in rounds of active learning. While XGBoost can still reach its highest performance after 6 rounds with only 10 data points per round, the linear regressors model show a poor performance with 10 data points per round. Note that the axis in linear regressors plot with 10 data points per round is different (smaller) than all the other plots. The boxplots with whisker length of 1.5, represent the minimum, 25th percentile (bottom bound of box), median (center of box), 75th percentile (upper bound of box), and maximum.

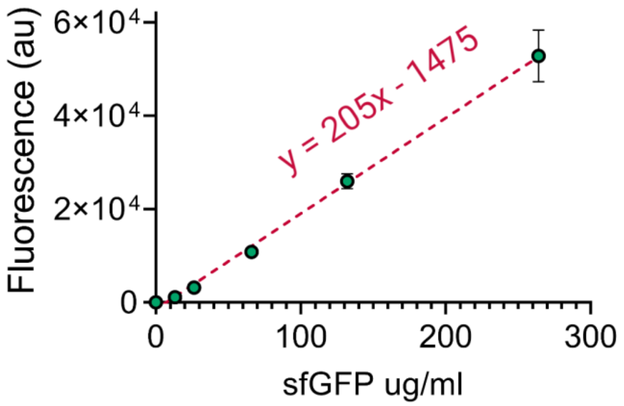

Supplementary Fig. 2: Standard curve for fluorescence readout of purified sfGfp (26806 Da) at the condition of the reference cell-free reaction. The experiment was performed in the same plate reader (Tecan Infinite 200 PRO, excitation/emission wavelengths of 485/528 nM and gain = 80). The average of measured Gfp fluorescence for the reference composition[8] is 3849.13 which corresponds to a concentration of 25.97 ug/ml (0.97 µM). This is the amount of sfGfp for the reference that we used to normalize all the data points in **Fig. 1e**. Quick math shows that the points with a yield of ~20 in **Fig. 1e** have a GPF production of ~0.97 $\times$ 20 = 19.4 µM. The plotted values are the average of triplicates (n=3 independent experiments), and the error bars represent the standard deviation. Source data are provided as a Source Data file.

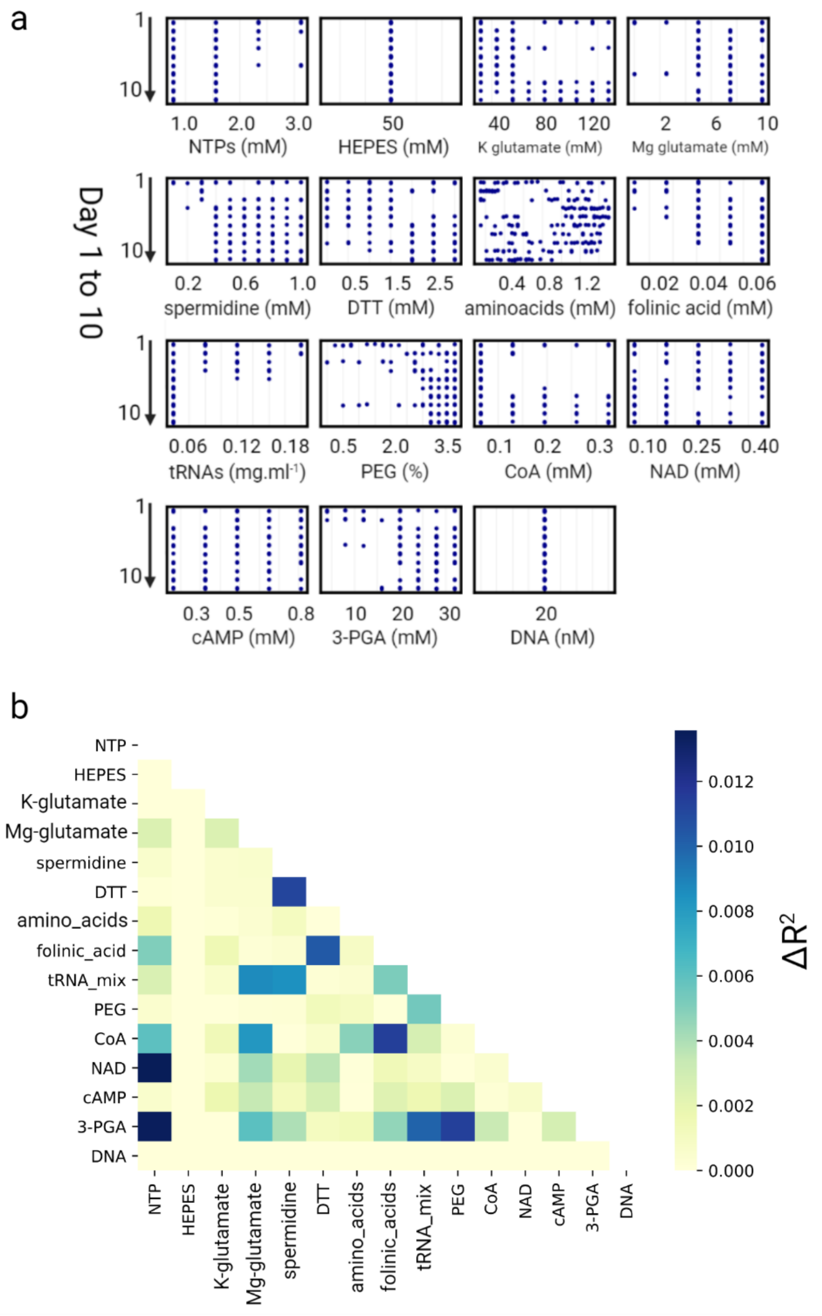

Supplementary Fig. 3: Concentration variations of each of the *E. coli* cell-free system factors from rounds (days) 1 to 10 of active learning (a), and mutual interactions between every two factors for active learning of the *E. coli* cell-free system (b). a: These plots show how the concentration of each factor varied from day 1 to 10 through suggestions of the model aiming to increase the yield. The features' importance (**Fig. 1f**), and yield dependencies (**Fig. 1g**) could be analyzed altogether with these plots. **b:** This plot shows the calculated mutual interactions (**Methods**) between every two factors, a useful analysis of the active learning dataset helping to study the system. This module is also integrated into the modular workflow.

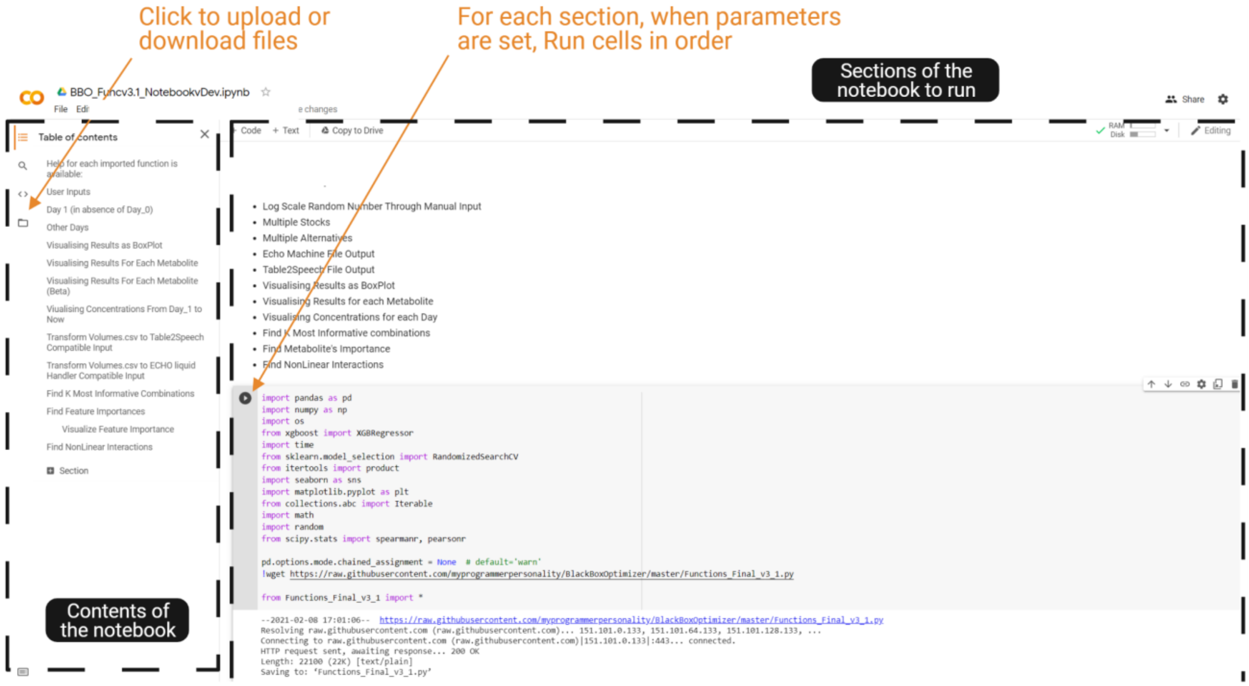

Supplementary Fig. 4: A screenshot of the Google Colab notebook for interacting with the workflow. At the left side of the page, the folder icon (shown by the arrow) is where files should be uploaded (Results) or downloaded (those that the user generated during the usage such as Volume, Concentration, Features' importance, K most informative, as well as figures as .png or .svg). When the run time is over, the user should reupload the files. The main body of the tool is where different cells (modules) of the code are accessible that should be run in order by clicking on the run icon (shown by the arrow). Except for the first round for which there is no results or input file, the files should be uploaded before starting to run the cells. For more details see **Code availability** to open (after making a personal copy) any of provided notebooks in your browser.

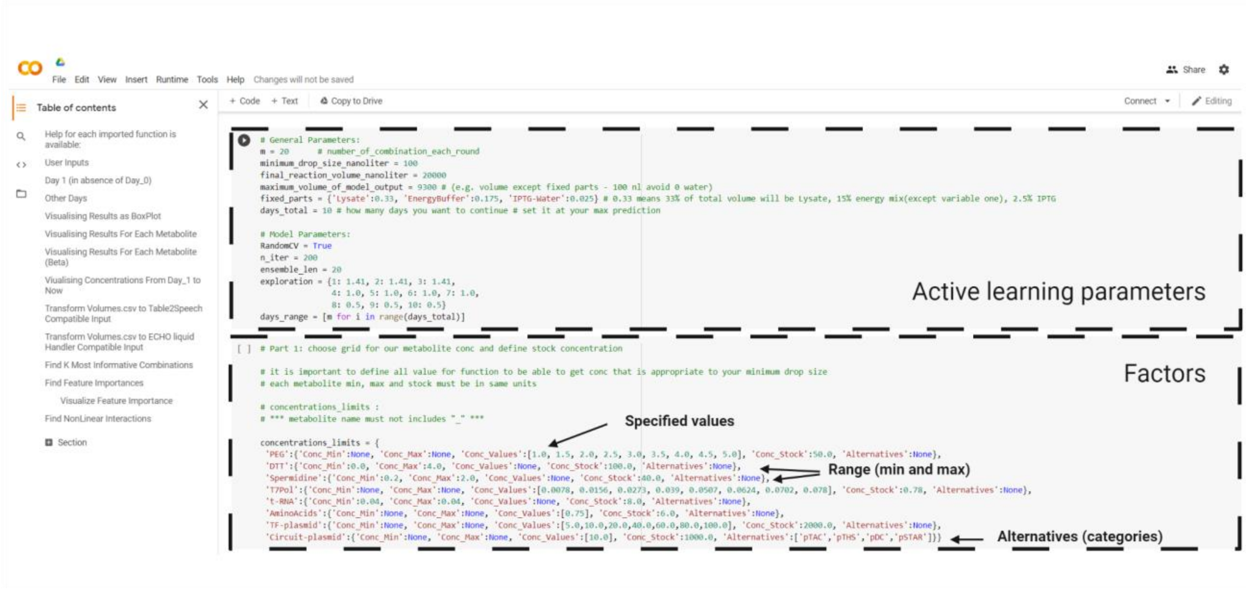

Supplementary Fig. 5: A screenshot of the sections of active learning parameters and factors. These two sections appear at the top of the notebook. These cells come after the sections for python classes/modules that should run beforehand. See **Supplementary Note 2** for detailed explanations.

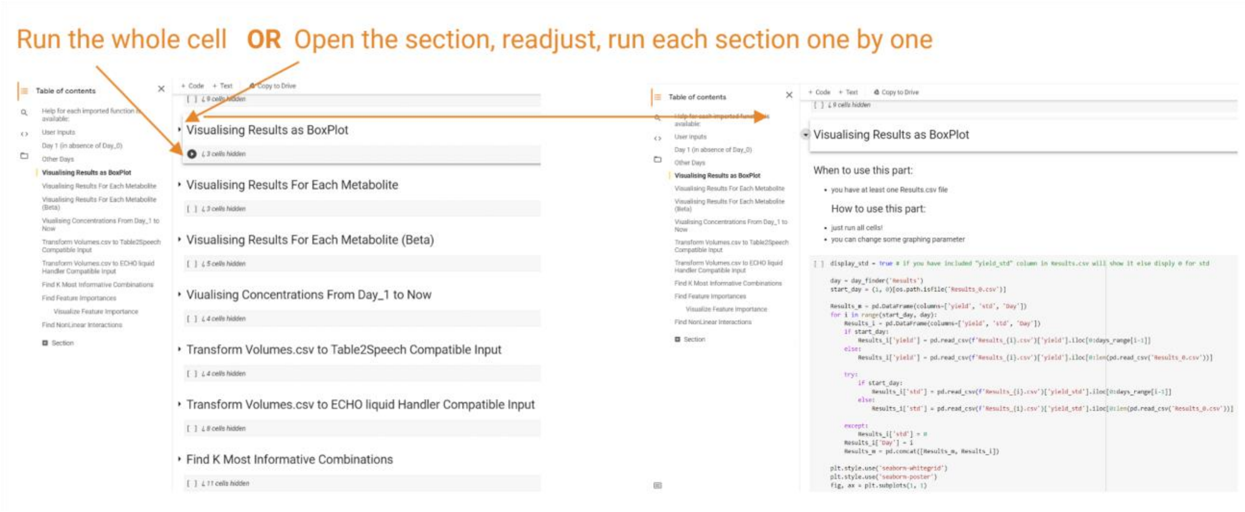

Supplementary Fig. 6: A screenshot of different modules of the workflow. The modules that a user aims to use can be run all at once or the user can open individual code sections/tabs and make changes for example in the plot size, color, etc.

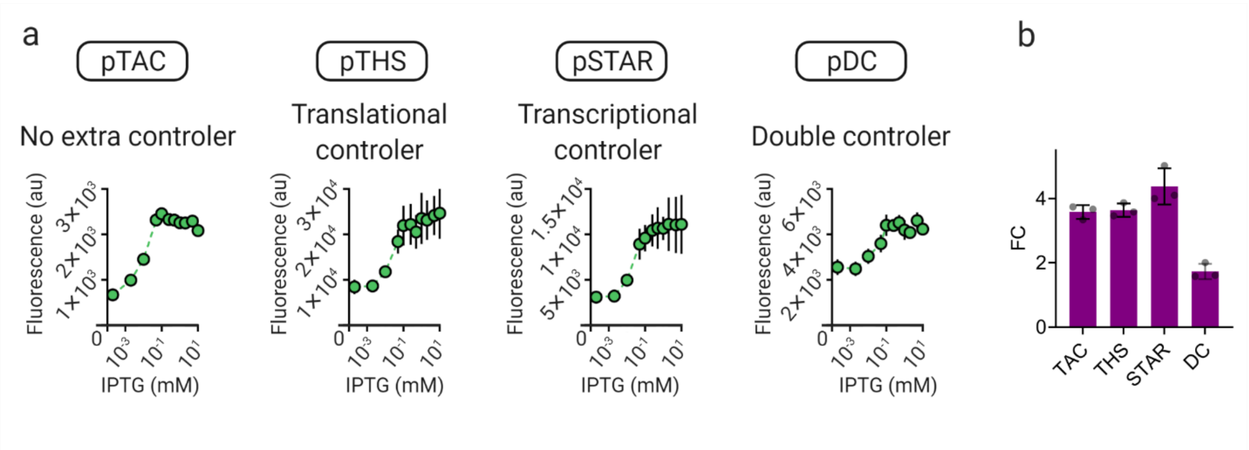

Supplementary Fig. 7: The behavior of *LacI* gene circuits in the cell-free protein expression system. These results presented in Greco *et al*.[10] as the "rate" in the protein production; however, here, we plotted the "amount" of protein produced after 6 h of incubation at 30 °C. **(a)** IPTG dose-response curve for SLC and MLC constructs. The plotted values are the average of triplicates (n=3 independent experiments), and the error bars represent the standard deviation. **(b)** The fold-change (FC) value of the plots in **(a)** between 0 and 10 mM of IPTG. The bars are the average of triplicates (n=3 independent experiments) and the error bars represent the standard deviation. Source data for **(a), (b)** are provided as a Source Data file

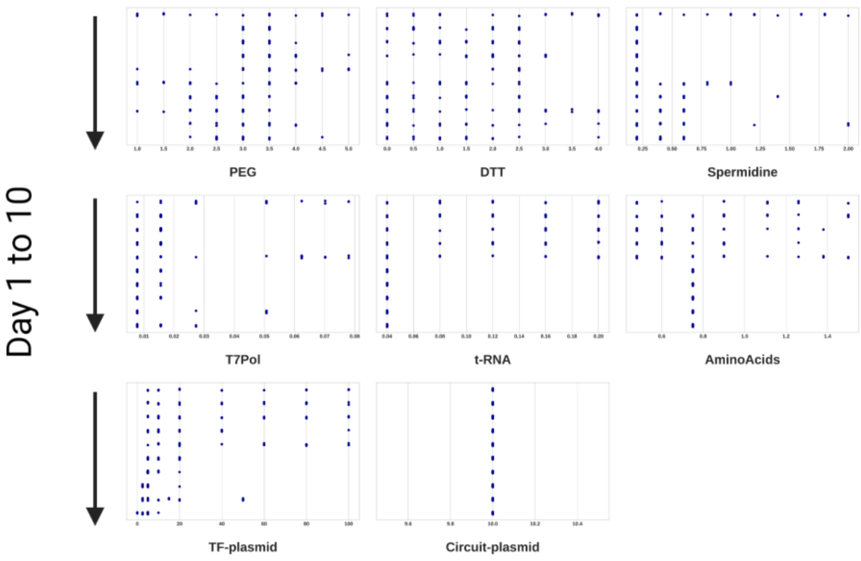

Supplementary Fig. 8: Variation of the concentration of factors in 10 rounds of active learning for *LacI* gene circuits optimization. These plots show suggestions of different concentrations of each factor by the model aiming to improve the objective function.

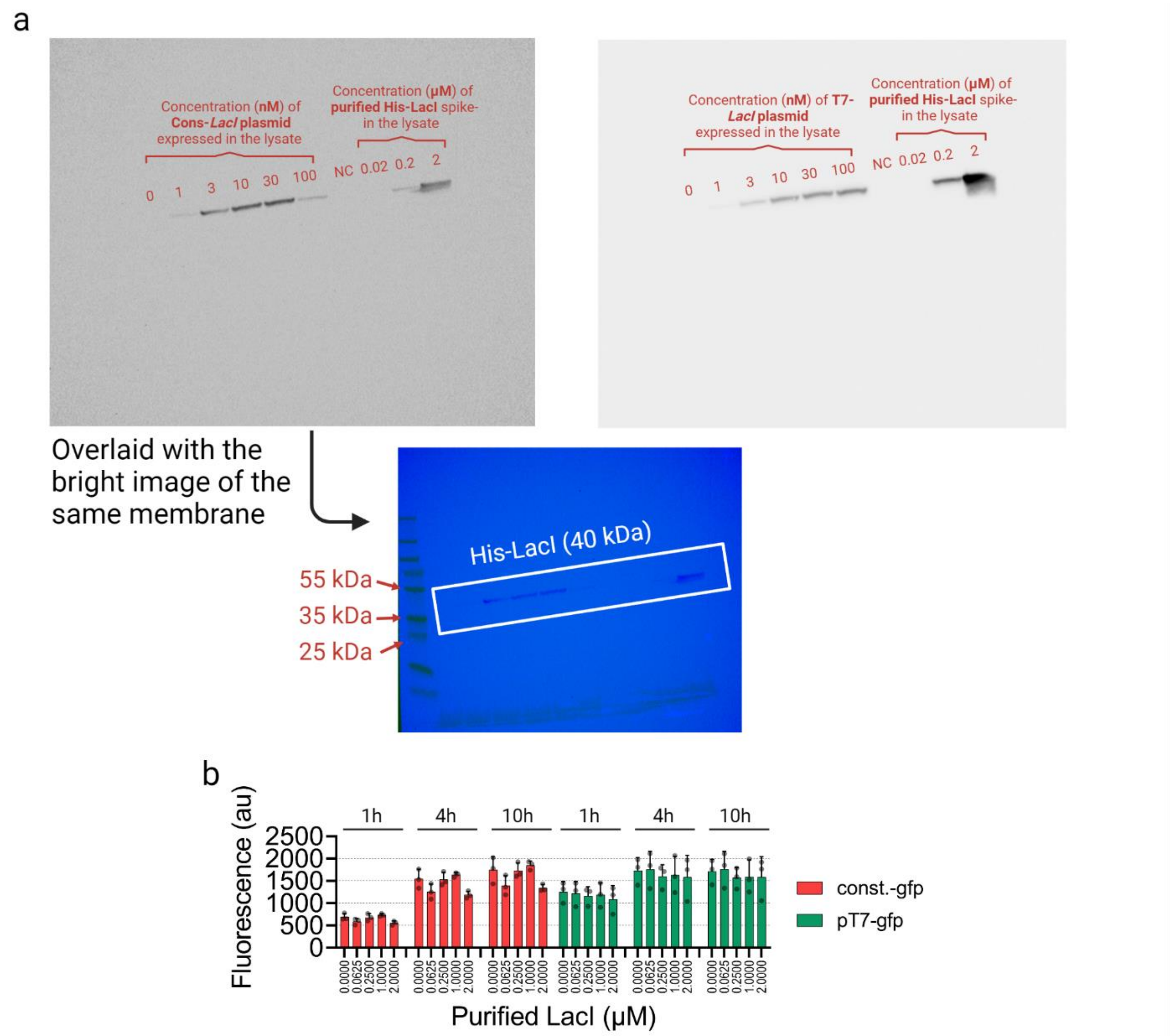

Supplementary Fig. 9: Gfp fluorescence level is affected by expression competition with *LacI*, not by interference by the LacI protein itself. a) Western chemiluminescence detection of LacI protein level produced in cell-free reactions starting with different concentrations of the *LacI* plasmid and purified LacI protein. The bottom blue image shows an overlay of the chemiluminescent signal and a bright image of the "same" membrane, where the ladder is visible. These experiments were performed as shown here with no replicates. **b)** Gfp fluorescence from cell-free reactions with different concentrations of purified LacI-6xHis protein included indicates that the LacI protein itself does not affect *Gfp* expression level at 2 μM and below (based on blots in **(a)** 2 μM is a concentration of LacI in the lysate more than what can be produced by expression from plasmids). The bars are the average of triplicates (n=3 independent experiments) and the error bars represent the standard deviation. Source data for **(b)** are provided as a Source Data file

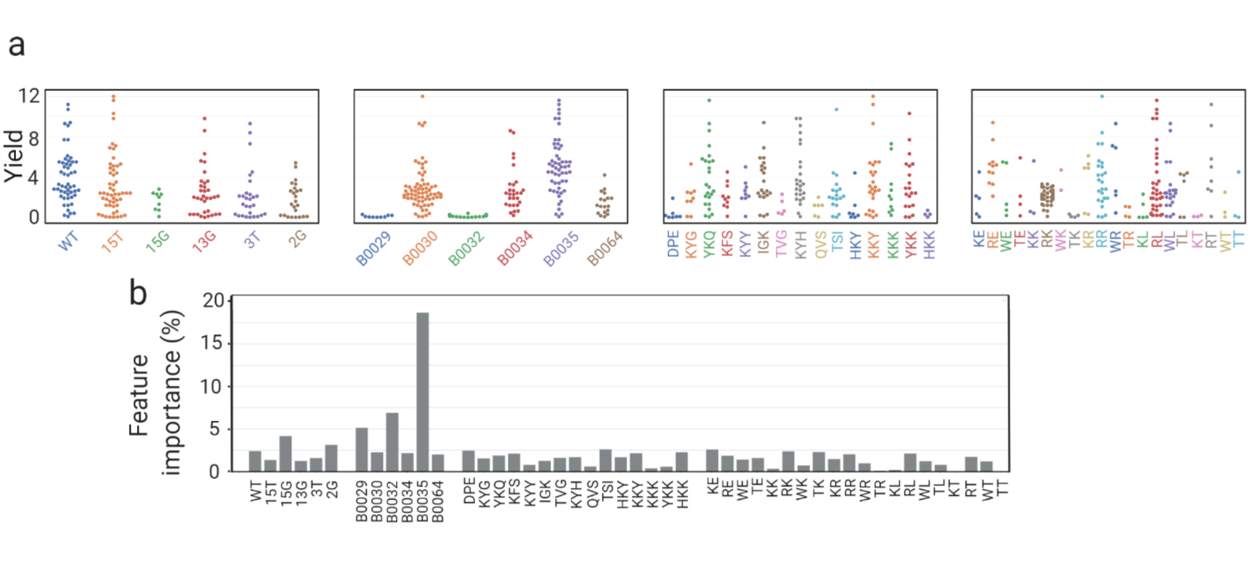

Supplementary Fig. 10: Analysis of the active learning data for the transcription and translation unit. (a) Distribution of alternative factors within the yield of 200 combinations. **(b)** The feature importance percentage of each condition for the model to assign predicted yield values.

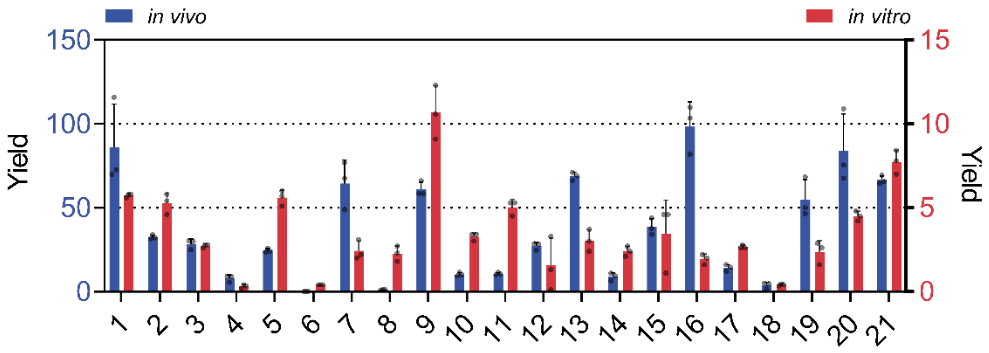

Supplementary Fig. 11: Cell-free versus *in vivo* yields of the 20 most informative combinations for the transcription & translation unit. The bar plot representation of results (average and standard deviation of triplicates, n=3 independent experiments) shown in **Fig. 4e**. Source data are provided as a Source Data file

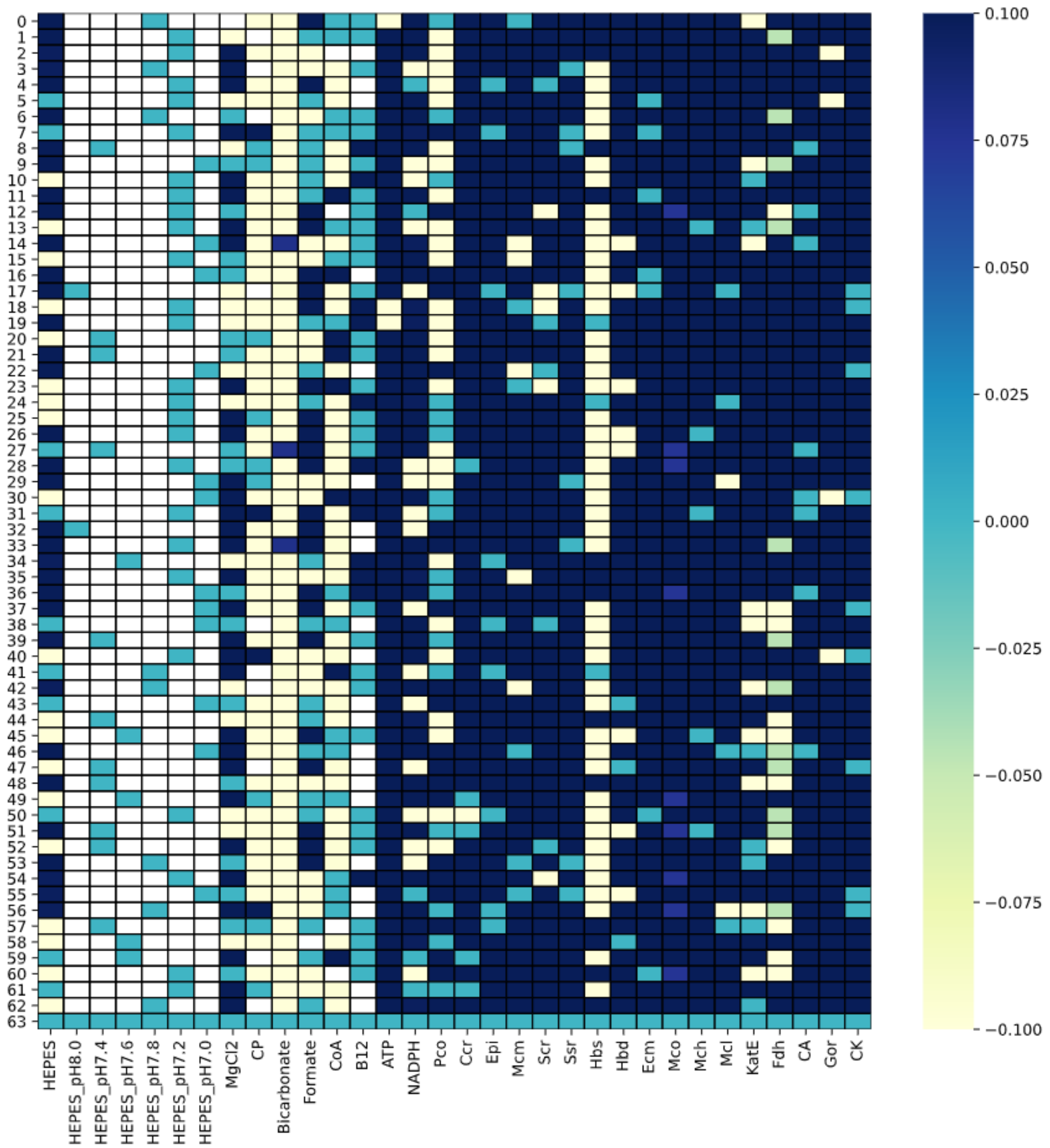

Supplementary Fig. 12: Scaled factors heatmap of top 10% yields in CETCH yield (glycolate, round 1-5) active learning. The concentration range for factors was scaled to the same range.

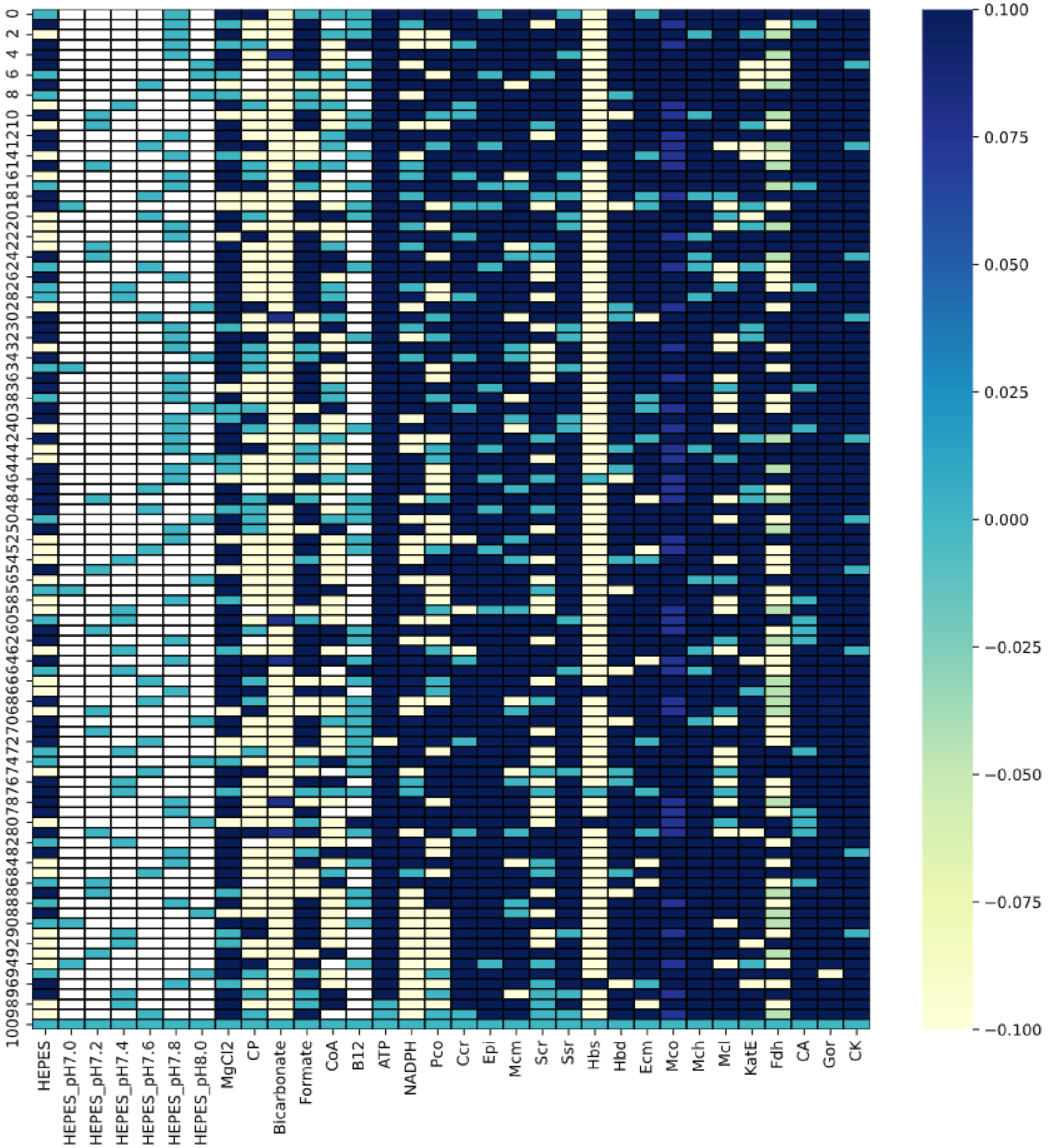

Supplementary Fig. 13: Scaled factors heatmap of top 10% yields in CETCH efficiency (glycolate concentration divided by the total enzyme concentration, round 1-8) active learning. The concentration range for factors was scaled to the same range.

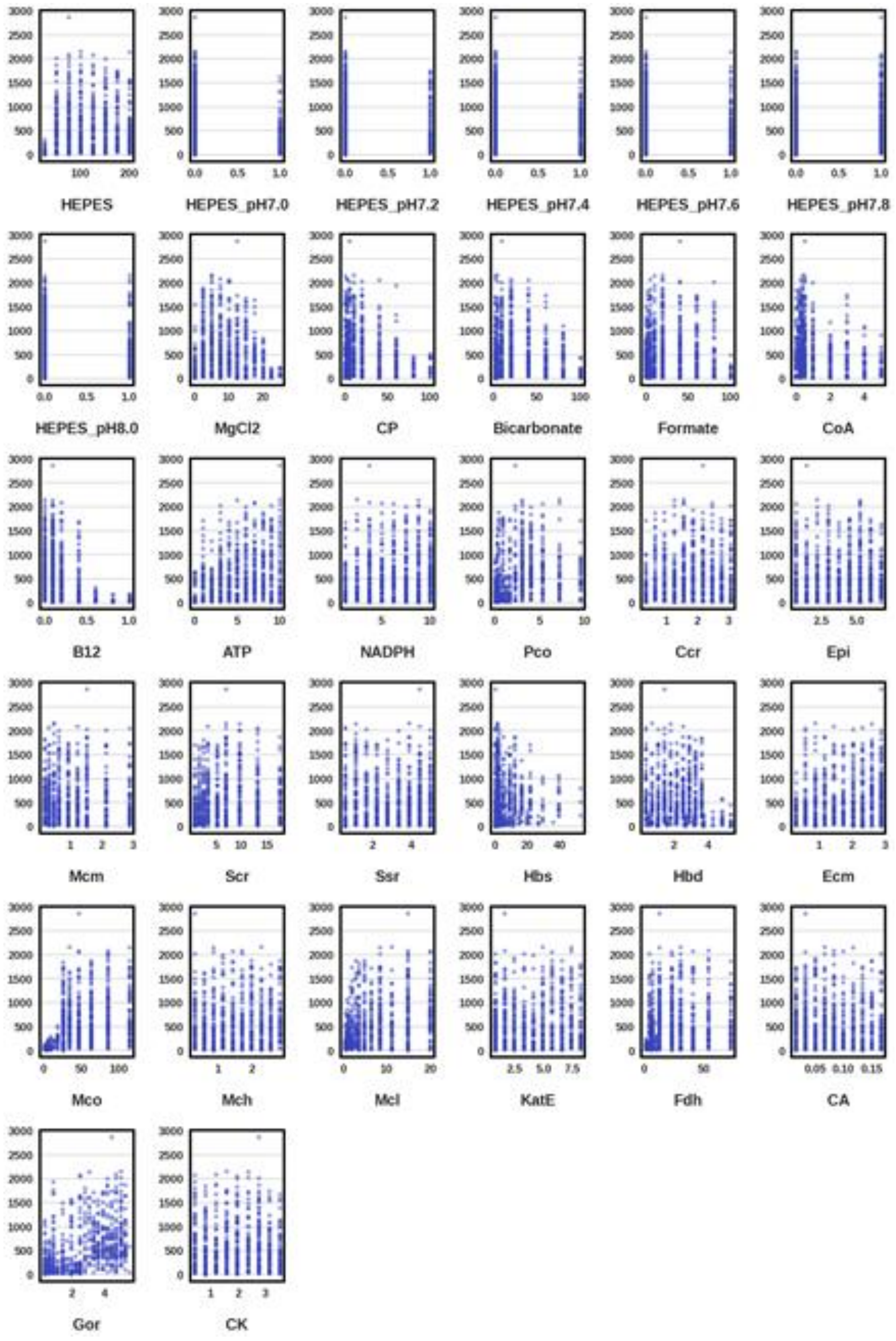

Supplementary Fig. 14: Distribution of measured yield (glycolate, round 1-5) values within the ranges of each factor. Distribution of all factors within the yield of 5 rounds.

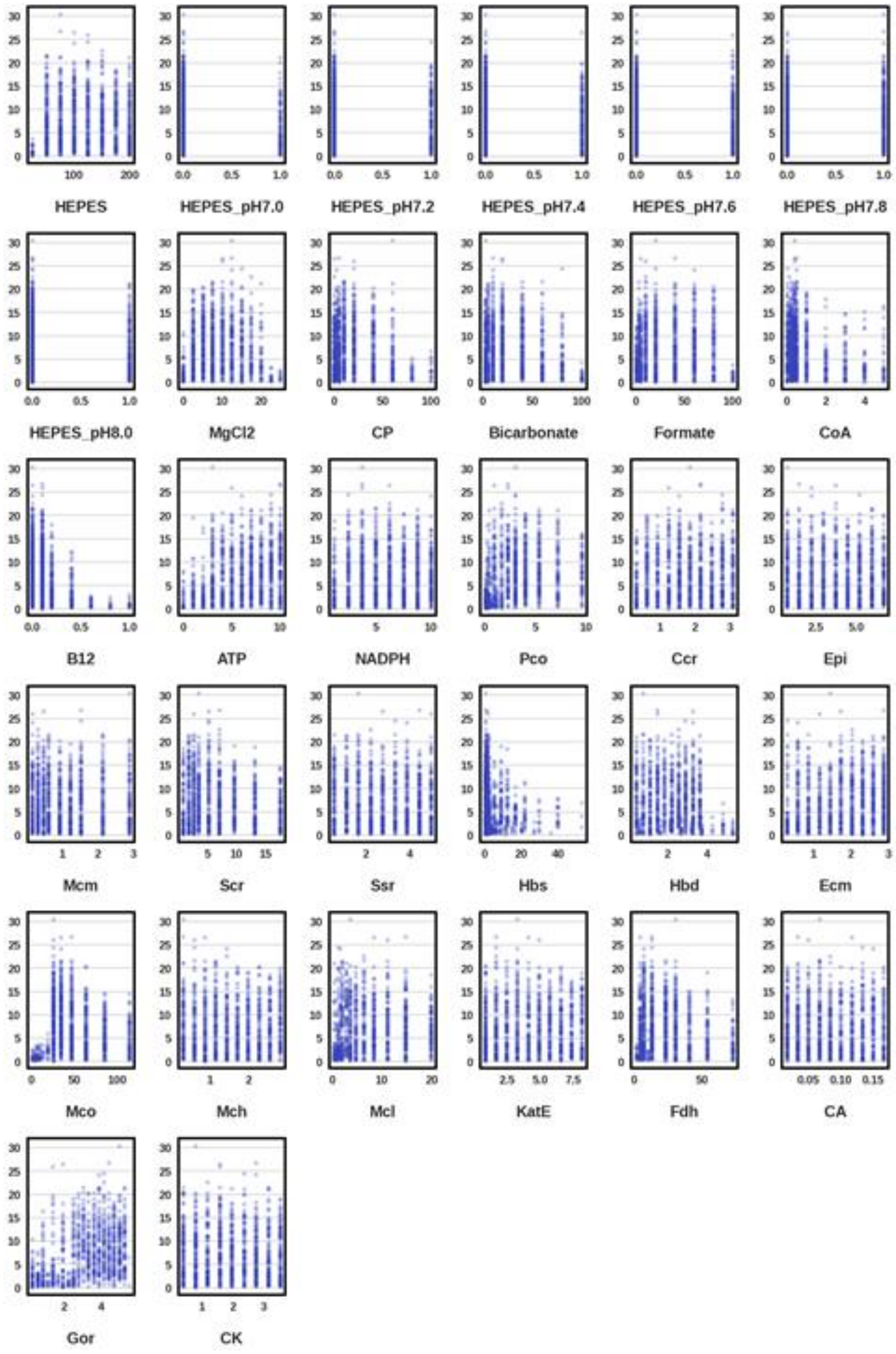

Supplementary Fig 15: Distribution of measured efficiency (glycolate concentration divided by the total enzyme concentration, round 1-8) values within the ranges of each factor. Distribution of all factors within the efficiency of 8 rounds.

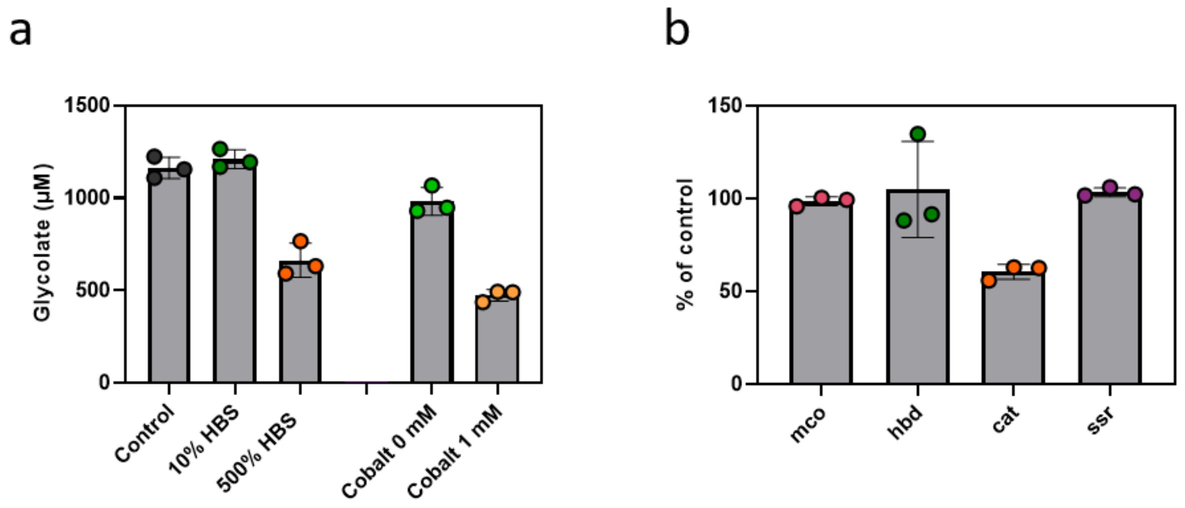

Supplementary Fig. 16: Assays for testing newly purified enzyme batches after round 2 and for testing different concentrations of hbs and cobalt. (a) The assays were done as described in material and methods (Assays for different concentrations of hbs and cobalt). The shown glycolate concentrations are from the sample taken after 180 min. The control contained 0.1 mM Coenzyme B_{12}, whereas the assays with 0 mM and 1 mM cobalt did not contain any Coenzyme B_{12}. **(b)** Test of old batches of mco, hbd, cat and ssr. After recognizing an increase in the product yield of our control after round two, we tested the old stocks of the enzymes which were purified freshly for round three in combination with the new enzymes. We could identify the old batch of catalase as the reason for the lower yield. The bars are the average of triplicates (n=3 independent experiments) and the error bars represent the standard deviation. Source data for **(a), (b)** are provided as a Source Data file.

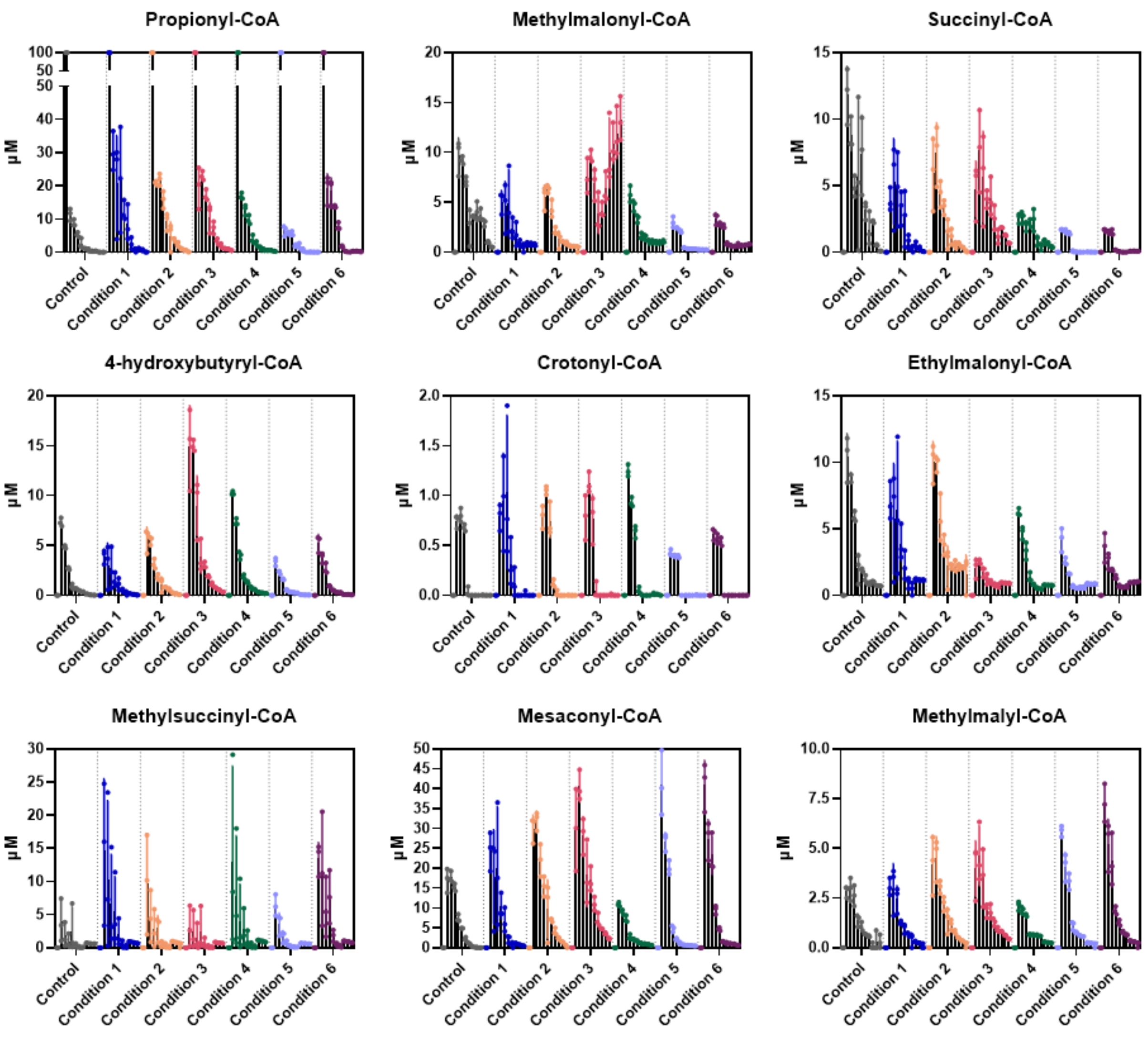

Supplementary Fig. 17: Quantified CoA-esters from selected assay conditions. The values for time point 0 are not measured, the reactions were started with 100 µM propionyl-CoA. The value of the black bars are the average of triplicates (n=3 independent experiments), and the error bars represent the standard deviation. For details see LC-MS analysis of CoA esters. Source data are provided as a Source Data file.

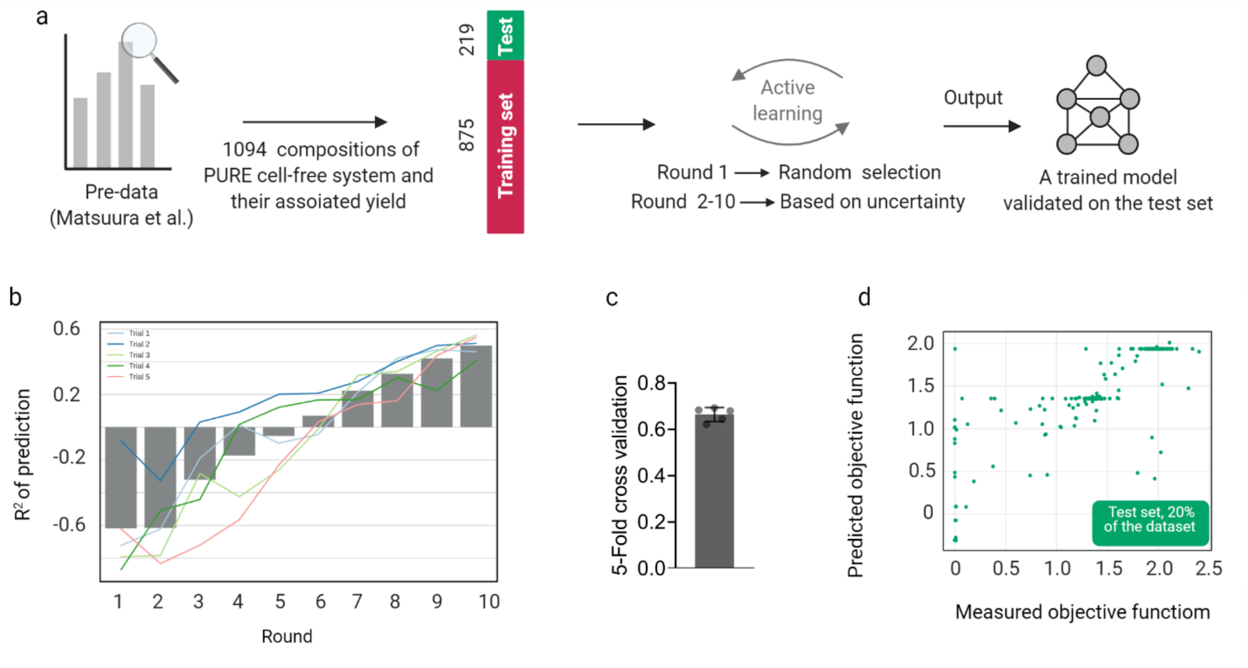

Supplementary Fig. 18: Application of METIS for the prediction of a PURE cell-free system dataset. (a) We took this dataset from a recent study in which they varied recombinant proteins and buffer compositions, adapted it as a Results.csv file. The dataset was divided into two subsets 80% for training and 20% for testing. (b) During 10 rounds of active learning, the model was assessed by the initial test set and the R^2 of the prediction improved over rounds. See **Supplementary Note 6** for a detailed explanation of the process. **(c)** 5-fold cross-validation to evaluate the average performance of the model on the whole dataset (The bar and the error bar represent the average and standard deviation, n=5 independent simulations). **(d)** a single round of validation of a model on the test set. The Google Colab Python notebook and all active learning data (combinations and yields) in this figure are available at https://github.com/amirpandi/METIS.

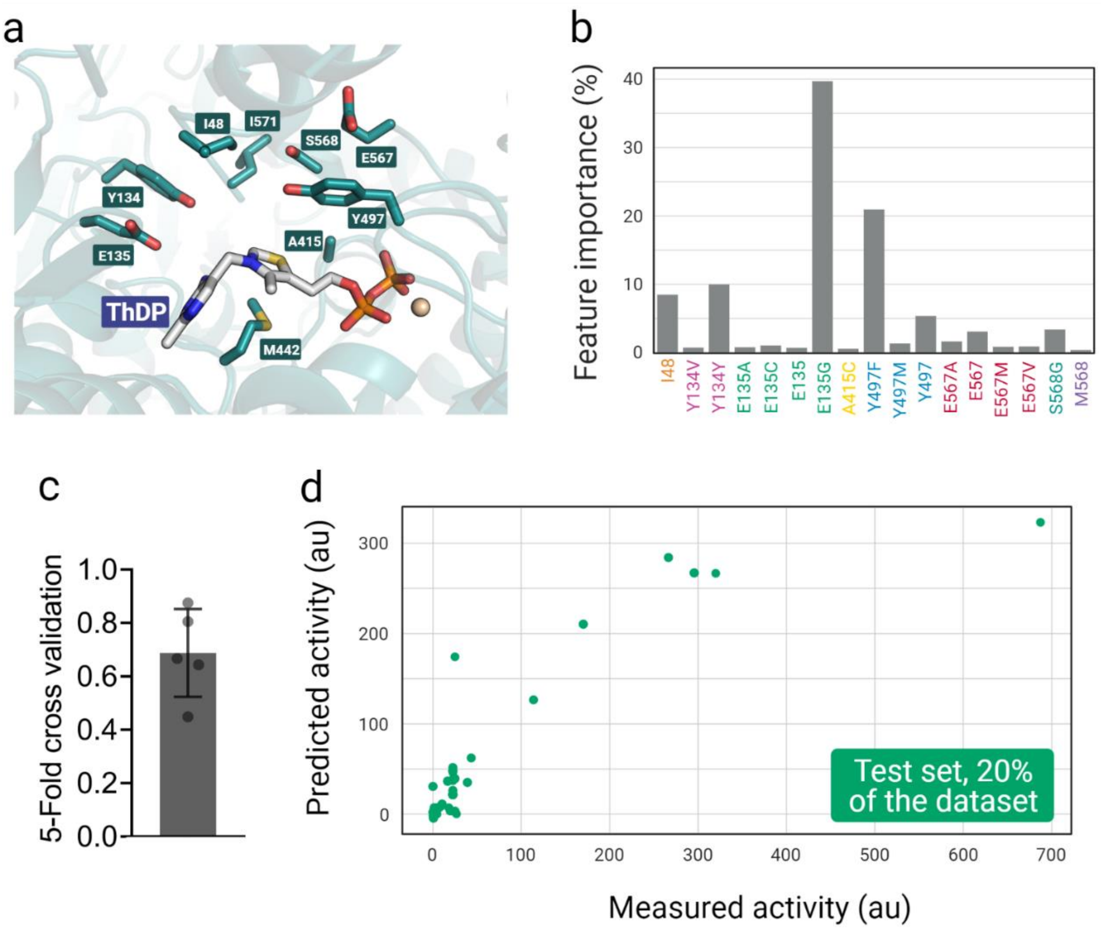

Supplementary Fig. 19: Simulations on a dataset of 847 mutants of oxalyl-CoA decarboxylase enzyme to showcase the application of METIS workflow for combinatorial enzyme engineering. (a) The active site of oxalyl-CoA decarboxylase with surrounding residues annotated and the covalently bound cofactor thiamine diphosphate (ThDP). **(b)** Calculated features' importance by the model on the imported dataset that define how big the effect of each condition is if the model suggests new amino acid combinations. **(c)** 5-fold cross-validation, a common approach to assess the prediction power of machine learning models (The bar and the error bar represent the average and standard deviation, n=5 independent simulations). **(d)** Predicted values on a single unseen test set (20% of the whole dataset) versus their measured values. (See also **Supplementary Note 7**). The Google Colab Python notebook and all active learning data (combinations and yields) in this figure are available at https://github.com/amirpandi/METIS. Source data for **(a), (e)** are provided as a Source Data file.

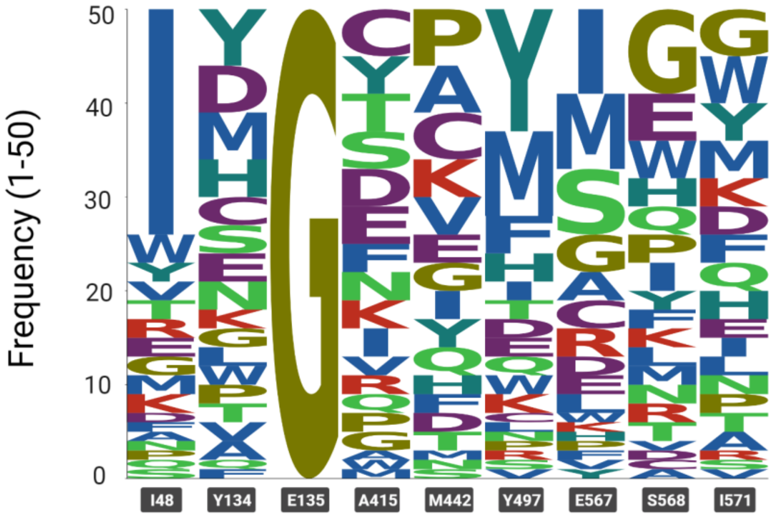

Supplementary Fig. 20: Distribution of amino acids in each position of the oxalyl-CoA decarboxylase active site predicted by the model out of 50 suggestions. This shows an example in case a user aims to synthesize or clone new enzyme sequences and test a round of combinatorial mutants.

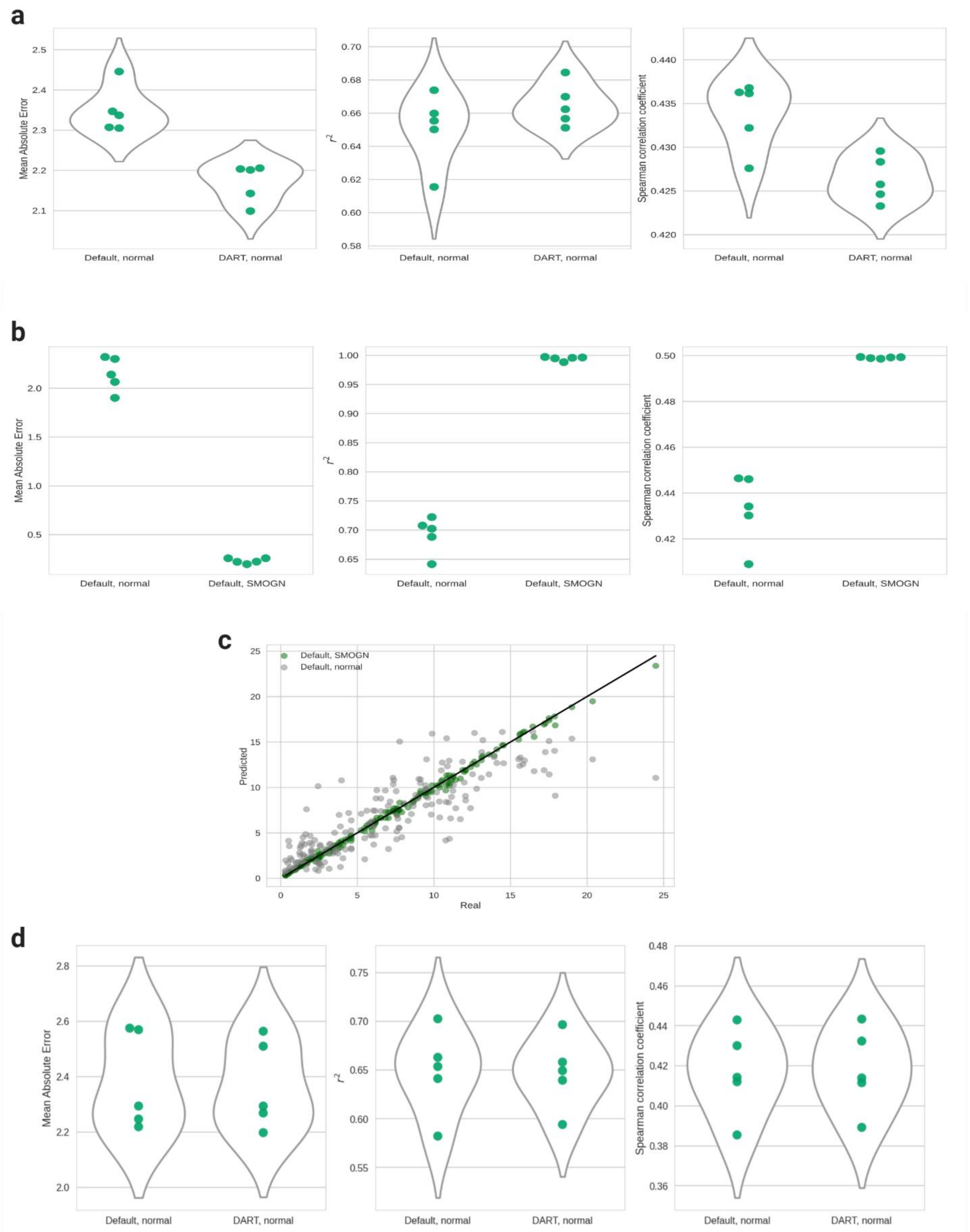

Supplementary Fig. 21: DART vs default (gbtree) XGBoost algorithms and SMOGN data preprocessing. The performance of two algorithm options on original and SMOTE oversampled data was compared using the same k-fold cross-validation setup on the CETCH cycle efficiency optimization dataset (1000 data points). For SMOGN comparison the entire dataset was used for k-fold cross-validation analysis, while the comparison between "gbtree" and "dart" algorithms occurred additionally a smaller dataset

(k-fold on 360 data points, rest – for validation). For the “dart” algorithm additional parameters were included into the parameter grid for optimization: rate_drop [0.02, 0.2], skip_drop [0.01, 0.1]. The resulting mean absolute error, r^2 and spearman correlation distributions are presented in **(a)** and **(b)**. Predictions for an exemplary test set from the k-fold validation experiment was additionally plotted against true values **(c)**. Active learning in biological applications is further known to suffer from overfitting that results from the small number of labeled samples[11]. To address this issue, we included the XGBoost implementation of DART algorithm to our workflow as an option. This algorithm introduces dropout – a widely used regularization method from the field of deep neural networks – boosted trees[12]. To additionally correct for the uneven data distribution that can occur at late stages of optimization, optional Synthetic Minority Over-Sampling Technique for Regression with Gaussian Noise (SMOGN)[13] input preprocessing was included into our workflow. This algorithm allows for synthetic oversampling of underrepresented datapoints based on the original dataset. As expected, the use of the DART algorithm did not significantly improve predictions for models trained on the large dataset (all 1000 data points) **(d)**. However, in case of the small dataset optimal parameters allowed for lowering of the prediction variance between cross-validation folds as well as MAE, thus suggesting overfitting reduction **(a)**. It is worth noting that the DART model training is slower due to the missing use of the prediction buffer and the optimization parameters need to be adapted for each data type. Thus, the overfitting optimization remains a question of gain versus time ratio for each experimental setup. As can be seen from the presented figures **(b)** additional data preprocessing using SMOGN significantly improved the performance of the respective models within all tested metrics especially in the high-yield range. This can be explained by the fact that the oversampling of this data resulted in model bias towards high yields. The overall increased performance accuracy in the low-yield regime additionally suggests that the balancing of the dataset improves the training of the model in general. However, it is important to note, that oversampling can result in an artificial bias towards a small range of yield increasing parameters and this way will counter the exploration of the algorithm. Thus, oversampling only makes sense if a reasonably large (minimum 10% of the entire dataset) and variable data subset in the high-yield regime is present in the dataset.

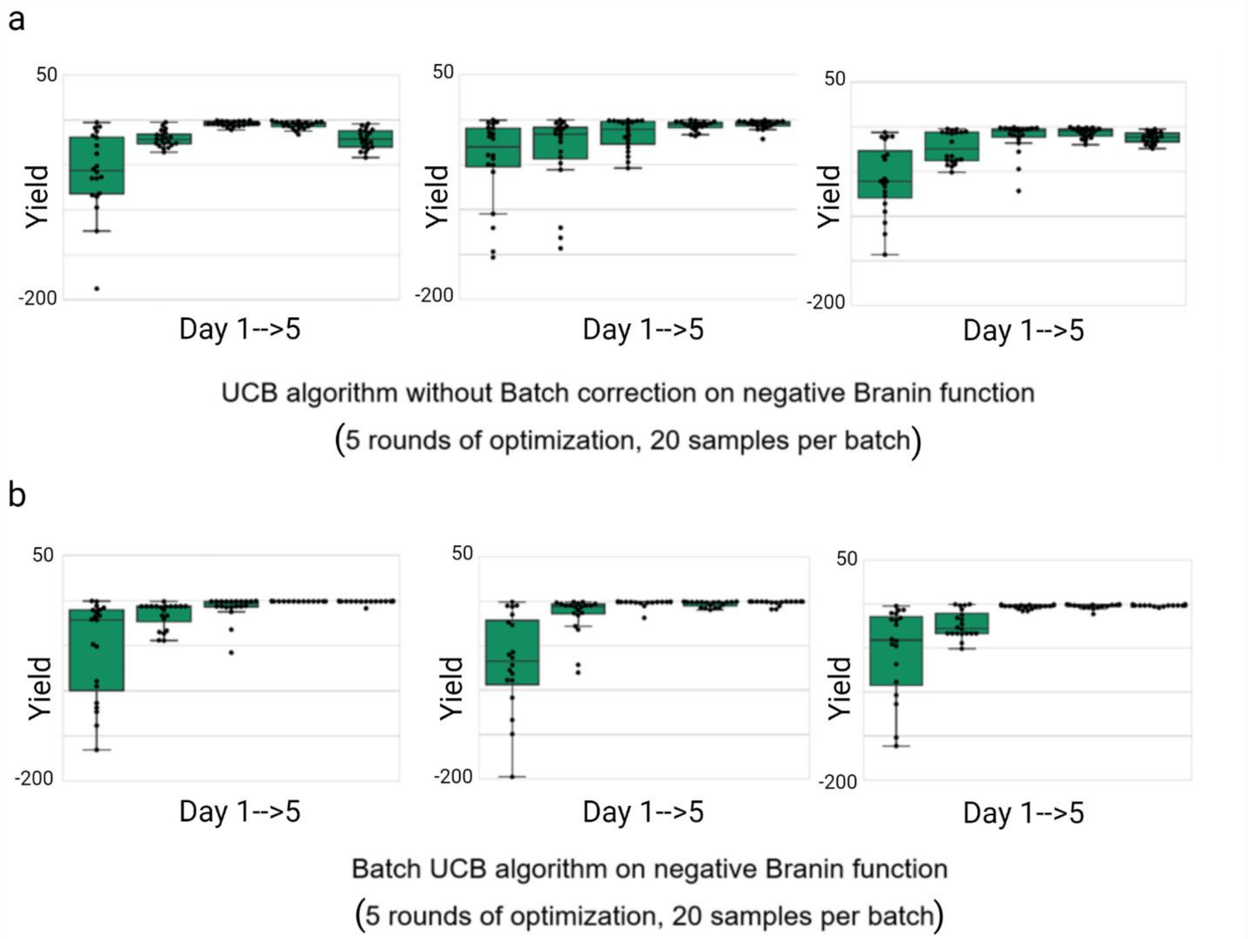

Supplementary Fig. 22: Batch Upper Confidence Bound (UCB) for scoring objective functions. We chose the Batch Upper Confidence Bound[14] algorithm because of its high compatibility and ease of implementation to the current version of METIS. This algorithm can be summarized as following to obtain a batch size of k: In each round the top UCB score sample is chosen, added to the training dataset with its predicted value, this process is repeated for k time. This algorithm avoids points similar to what already chosen in a batch since they are going to have low standard variation parameters in UCB score. We evaluated the performance of the Batch UCB algorithm **(b)** compared to UCB algorithm **(a)** on a test function (Branin Function[15]). Three independent simulations were repeated and plotted in **(a)** and **(b)**. The Batch UCB algorithm shows a performance slightly better than UCB and since it can be costly in term of time, we added it as an optional setting to METIS.

Supplementary References

1. Sommer, C. & Gerlich, D. W. Machine learning in cell biology - teaching computers to recognize phenotypes. *J. Cell Sci.* **126**, 5529–5539 (2013).
2. Borkowski, O. *et al.* Large scale active-learning-guided exploration for in vitro protein production optimization. *Nat. Commun.* **11**, 1–8 (2020).
3. Matsuura, T., Kazuta, Y., Aita, T., Adachi, J. & Yomo, T. Quantifying epistatic interactions among the components constituting the protein translation system. *Mol. Syst. Biol.* **5**, 297 (2009).
4. Yang, K. K., Wu, Z. & Arnold, F. H. Machine-learning-guided directed evolution for protein engineering. *Nat. Methods* **16**, 687–694 (2019).
5. Wittmann, B. J., Johnston, K. E., Wu, Z. & Arnold, F. H. Advances in machine learning for directed evolution. *Curr. Opin. Struct. Biol.* **69**, 11–18 (2021).
6. Wittmann, B. J., Yue, Y. & Arnold, F. H. Machine Learning-Assisted Directed Evolution Navigates a Combinatorial Epistatic Fitness Landscape with Minimal Screening Burden. *Cold Spring Harbor Laboratory* 2020.12.04.408955 (2020) doi:10.1101/2020.12.04.408955.
7. Nattermann, M. *et al.* Engineering a Highly Efficient Carboligase for Synthetic One-Carbon Metabolism. *ACS Catal.* **11**, 5396–5404 (2021).
8. Sun, Z. Z. *et al.* Protocols for implementing an Escherichia coli based TX-TL cell-free expression system for synthetic biology. *J. Vis. Exp.* e50762 (2013).
9. Ribosome Binding Sites/Prokaryotic/Constitutive/Community Collection. https://parts.igem.org/Ribosome_Binding_Sites/Prokaryotic/Constitutive/Community_Collection.
10. Greco, F. V., Pandi, A., Erb, T. J., Grierson, C. S. & Gorochowski, T. E. Harnessing the central dogma for stringent multi-level control of gene expression. *Nat. Commun.* **12**, 1738 (2021).
11. Yang, Y., Ma, Z., Nie, F., Chang, X. and Hauptmann, A.G. Multi-class active learning by uncertainty sampling with diversity maximization. *International Journal of Computer Vision*, 113(2), 113-127 (2015)
12. Vinayak, R.K. and Gilad-Bachrach, R. Dart: Dropouts meet multiple additive regression trees. *in Artificial Intelligence and Statistics,* 489-497 (2015).
13. Branco, P., Torgo, L. and Ribeiro, R.P. SMOGN: a pre-processing approach for imbalanced regression. *in First international workshop on learning with imbalanced domains: Theory and applications*, 36-50 (2017).
14. Hunt, N., Batch Bayesian optimization. (Massachusetts Institute of Technology, 2020).
15. Virtual library of simulation experiments: Test functions and datasets, https://www.sfu.ca/~ssurjano/branin.html.
