# Supplementary material for: A versatile active learning workflow for optimization of genetic and metabolic networks
Source: Nat Commun. 2022 Jul 5;13:3876. doi: 10.1038/s41467-022-31245-z (PMC9256728; doi:10.1038/s41467-022-31245-z)
Supplement: Supplementary file 3 — Reporting Summary [file 41467_2022_31245_MOESM3_ESM.pdf]

nature portfolio

Corresponding author(s): Tobias J. Erb, Amir Pandi

Last updated by author(s): Jun 9, 2022

Reporting Summary

Nature Portfolio wishes to improve the reproducibility of the work that we publish. This form provides structure for consistency and transparency in reporting. For further information on Nature Portfolio policies, see our Editorial Policies and the Editorial Policy Checklist.

Statistics

For all statistical analyses, confirm that the following items are present in the figure legend, table legend, main text, or Methods section.

n/a	Confirmed	
☐	☒	The exact sample size (*n*) for each experimental group/condition, given as a discrete number and unit of measurement
☐	☒	A statement on whether measurements were taken from distinct samples or whether the same sample was measured repeatedly
☐	☒	The statistical test(s) used AND whether they are one- or two-sided<br>*Only common tests should be described solely by name; describe more complex techniques in the Methods section.*
☒	☐	A description of all covariates tested
☒	☐	A description of any assumptions or corrections, such as tests of normality and adjustment for multiple comparisons
☐	☒	A full description of the statistical parameters including central tendency (e.g. means) or other basic estimates (e.g. regression coefficient) AND variation (e.g. standard deviation) or associated estimates of uncertainty (e.g. confidence intervals)
☒	☐	For null hypothesis testing, the test statistic (e.g. *F*, *t*, *r*) with confidence intervals, effect sizes, degrees of freedom and *P* value noted<br>*Give P values as exact values whenever suitable.*
☒	☐	For Bayesian analysis, information on the choice of priors and Markov chain Monte Carlo settings
☒	☐	For hierarchical and complex designs, identification of the appropriate level for tests and full reporting of outcomes
☒	☐	Estimates of effect sizes (e.g. Cohen's *d*, Pearson's *r*), indicating how they were calculated

Our web collection on statistics for biologists contains articles on many of the points above.

Software and code

Policy information about availability of computer code

Data collection	Fluorescence data was collected on Tecan Infinite 200 PRO plate reader using the dedicated software. All glycolate and CoA esters were collected on a triple quadrupole mass spectrometer (Agilent Technologies 6495 Triple Quad LC/MS).
Data analysis	Data was analysed using MS Excel, GraphPad Prism, and custom Python scripts (available at https://github.com/amirpandi/METIS) and Agilent Mass Hunter Workstation Software (QQQ) 10.0 for LC-MS data.

For manuscripts utilizing custom algorithms or software that are central to the research but not yet described in published literature, software must be made available to editors and reviewers. We strongly encourage code deposition in a community repository (e.g. GitHub). See the Nature Portfolio guidelines for submitting code & software for further information.

Data

Policy information about availability of data

All manuscripts must include a data availability statement. This statement should provide the following information, where applicable:

- Accession codes, unique identifiers, or web links for publicly available datasets
- A description of any restrictions on data availability
- For clinical datasets or third party data, please ensure that the statement adheres to our policy

The source data underlying Figures 3f-i, 4a, e, and 5h-l and Supplementary Figures 2, 7a, b, 9b, 11, 16a, b are provided as a Source Data file. All active learning data (combinations and yields) are available along with Google Colab Python notebook of each application on GitHub, https://github.com/amirpandi/METIS. Primers used for transcription and translation units (Fig. 4) are provided in Supplementary Table 8, 9.

Field-specific reporting

Please select the one below that is the best fit for your research. If you are not sure, read the appropriate sections before making your selection.

- [x] Life sciences
- [ ] Behavioural & social sciences
- [ ] Ecological, evolutionary & environmental sciences

For a reference copy of the document with all sections, see nature.com/documents/nr-reporting-summary-flat.pdf

Life sciences study design

All studies must disclose on these points even when the disclosure is negative.

Sample size

No sample size calculation was performed. According to many other similar works in the field, a sample size of three was chosen for all experiments.

Data exclusions

No data was excluded from the analyses

Replication

For in vivo experiments, replicates were run simultaneously from three separate colonies. For cell-free/in vitro experiments, replicates were run simultaneously by independently loading three separate reactions on the plate. All attempts at replication were successful.

Randomization

We did not have any participants and medical or environmental samples in this study. All samples used in this study were prepared by the investigation in the laboratory or suggested by machine learning algorithms (optimization of known biological systems), hence, no randomization was needed.

Blinding

Blinding of the investigation is not relevant to this study because the experiments were performed with the purpose of the optimization of known biological systems. All samples with proper controls were prepared by the investigation in the laboratory or suggested by machine learning algorithms.

Reporting for specific materials, systems and methods

We require information from authors about some types of materials, experimental systems and methods used in many studies. Here, indicate whether each material, system or method listed is relevant to your study. If you are not sure if a list item applies to your research, read the appropriate section before selecting a response.

Materials & experimental systems

n/a	Involved in the study	
☐	☒	Antibodies
☒	☐	Eukaryotic cell lines
☒	☐	Palaeontology and archaeology
☒	☐	Animals and other organisms
☒	☐	Human research participants
☒	☐	Clinical data
☒	☐	Dual use research of concern

Methods

n/a	Involved in the study	
☒	☐	ChIP-seq
☒	☐	Flow cytometry
☒	☐	MRI-based neuroimaging

Antibodies

Antibodies used

The monoclonal antibody against LacI, clone 9A5 (Sigma-Aldrich #05-503-I) and an anti-mouse HRP-conjugated secondary antibody (Invitrogen #31430)

Validation

The monoclonal antibody against LacI (Sigma-Aldrich #05-503-I) is a commonly used antibody in Molecular Biology and it has been validated by the provider with Western Blot in Rosetta BL21 (DE3) E. Coli cell lysate. Additionally, in our work with negative control and in vitro experiment with different concentration of LacI, we observed the expected results from the antibody.
